# Meta-analysis of functional genomics studies reveals conserved cellular pathways required by viruses of pandemic concern

**DOI:** 10.1099/acmi.0.001167.v3

**Published:** 2026-06-19

**Authors:** Aaron M. Brice, Alessandro T. Caputo, Cameron R. Stewart

**Affiliations:** 1 Commonwealth Scientific and Industrial Research Organisation, Health & Biosecurity at the Australian Centre of Disease Preparedness, Geelong, Victoria, Australia; 2 Commonwealth Scientific and Industrial Research Organisation, Manufacturing, Parkville, Victoria, Australia

**Keywords:** functional genomics, host-directed antivirals, meta-analysis, pandemic preparedness, virus–host interface

## Abstract

The coronavirus disease 2019 pandemic illustrated the need to develop medical countermeasures against emerging infectious diseases. As viruses rely on cellular machinery for replication, host-directed antivirals (HDAs) may complement conventional antiviral strategies in a manner that offers broad-spectrum efficacy, including against novel viruses, and a potentially higher barrier to viral escape. Despite their potential, HDAs are under-represented as therapeutics, partly due to a lack of consensus on druggable host targets. To address this, we have performed a meta-analysis of 62 functional genomics studies involving viral families of pandemic concern, including *Arenaviridae*, *Coronaviridae*, *Filoviridae*, *Flaviviridae*, *Orthomyxoviridae*, *Paramyxoviridae*, *Phenuiviridae*, *Picornaviridae*, *Poxviridae* and *Togaviridae*. Using a RRA and protein–protein interaction network approach, host factors and cellular processes required by multiple virus families were identified, including the V-type ATPase complex, glycosaminoglycan synthesis, Golgi trafficking and endoplasmic reticulum membrane protein insertion. These pathways include those with known relevance to infection by some viruses while providing novel insights into the life cycles of others. Therapeutic targeting of these top ranked host factors is also discussed, with several already possessing small-molecule inhibitors, highlighting their therapeutic potential. Antivirals are an essential component of medical countermeasures against viral disease and fulfil a complementary role to vaccines. Importantly, they may provide the only therapeutic option for pathogens lacking effective vaccines or for individuals unable to be vaccinated. This analysis furthers our understanding of the virus–host interface and nominates cellular targets for the future development of HDAs as medical countermeasures for pandemic resilience.

## Data Summary

All data used and produced in this manuscript are available in the supplementary files.

## Introduction

The World Health Organization (WHO) and other national organizations have curated lists of priority pathogens to guide research and development for potential future epidemics and pandemics [1,3]. *Arenaviridae*, *Coronaviridae*, *Filoviridae*, *Flaviviridae*, *Hantaviridae*, *Nairoviridae*, *Orthomyxoviridae*, *Paramyxoviridae*, *Peribunyaviridae*, *Phenuiviridae*, *Picornaviridae*, *Poxviridae* and *Togaviridae* viral families are classified as high-risk families due to their virulence, transmissibility and lack of medical countermeasures (summarized in Table S1, available in the online Supplementary Material) [1,3]. Notably, only a small number of viruses from these families have approved vaccines, therapeutic monoclonal antibodies (mAbs) or direct-acting antivirals (DAAs). Concerns over the efficacy of existing countermeasures are compounded by virus evolution that can render existing countermeasures ineffective. For example, substantial variance in vaccine-mediated protection has been observed for severe acute respiratory syndrome coronavirus-2 (SARS-CoV-2) vaccines between variants during the course of the pandemic, with overall effectiveness ranging between 88.0% for Alpha and as low as 55.9% for Omicron [4]. Escape of SARS-CoV-2 from mAb-based therapies has also been observed, including combinatorial therapy [4,6]. The efficacy of existing therapeutic strategies against emerging viruses and strains therefore remains uncertain.

Viruses rely on the cellular host machinery for their replication and viruses within the same family often exploit the same host factors for replication [7,9]. Host-directed antivirals (HDAs) could therefore offer a greater capacity for broad-spectrum efficacy, including against novel viruses [10]. HDAs may also provide a higher barrier for the development of viral escape, requiring the use of an alternative host factor or more efficient utilization of lower concentrations of the HDA target [11]. These have been observed in cell culture models but required a considerably longer period of exposure compared to DAAs [10,11]. Despite these points, the number of HDAs currently approved for clinical use is limited. Most are immunomodulators, predominantly IFNs, that boost immune clearance of viruses and are prescribed for chronic infections such as hepatitis C virus (HCV) and human immunodeficiency virus-1 (HIV-1) [11]. HDAs can also impact cellular processes more generally to inhibit infection, such as the guanosine analogue, ribavirin. The mechanism of action of ribavirin is not fully understood, but it exhibits both modulation of host gene expression (HDA function) and viral polymerase inhibition (DAA function) [12,13]. Ribavirin was primarily approved for treatment of chronic HCV infection, in combination with IFNs, but has been replaced by safer and more efficacious DAAs [14]. Limited evidence of efficacy against other viruses and significant side effects have restricted its use, except on compassionate grounds for when no other therapies are available [13,15,19]. Other HDAs are topical treatments for skin infections, including podophyllin and sinecatechins, used to treat warts caused by human papillomavirus, and docosanol, for the treatment of herpes simplex virus-1 infections [20,21]. The only clinically available HDA targeting a specific host factor required for virus infection is maraviroc, which targets CCR5, the entry receptor for HIV-1 [22].

Development of targeted HDAs requires knowledge of the host factors necessary for virus replication. Functional genomics is an integral tool to identify these host factors, enabling large-scale screens to be performed in a high-throughput and unbiased manner. This approach involves increasing (gain-of-function) or reducing (loss-of-function) expression of each gene in the host genome to assess their impact on virus replication. Readouts can include virus-induced cell death (for cytopathic viruses) or measurement of virus replication using tools such as immunofluorescence and reporter viruses [23]. Early functional genomics studies utilized RNA interference technology to induce the degradation of specific mRNA to inhibit protein expression [24]. The development of clustered regularly interspaced short palindromic repeats (CRISPR) technology has streamlined functional genomic screening by enabling targeted and permanent knockout of specific genes in a pooled manner, an approach termed genome-wide CRISPR-Cas9 functional knockout screening (GeCKO). A third, less common technique is gene-trapping whereby lentiviruses are used to induce insertional mutations throughout the genome to disrupt gene expression. This is similar to GeCKO but the insertions are random and unguided [25].

Although genome-wide studies are a powerful tool to identify virus–host factors, disparate results are often observed between similar studies, potentially due to differences in experimental protocols (e.g. virus strain, cell type, infectious dose, etc.). Meta-analyses can aid in unifying these datasets to elucidate the best candidates, but these are often limited to a single virus or several closely related viruses within the same genus or family (for example [26,28]). With the aim to identify candidates for the development of broad-spectrum HDAs, presented here is a meta-analysis of published genome-wide loss-of-function screens investigating multiple virus families of pandemic concern. This analysis identified key host factors and cellular pathways involved in infection both within and between virus families and represents a resource to progress research into HDAs and the development of countermeasures against current and novel pandemic viruses.

## Methods

### Literature search

A list of virus families of pandemic concern was developed based on reports released by the WHO [3], Commonwealth Scientific and Industrial Research Organisation (CSIRO) [1] and National Institute of Allergy and Infectious Diseases (NIAID) [2]. Genera and species for these virus families were obtained from the International Committee on Taxonomy of Viruses (ICTV; Master Species List #39, released July 2023) [29]. The species list was filtered based on those viruses known to infect humans using Virus–Host DB [30]; this search was performed on 3 April 2024. A literature search was performed using PubMed and Scopus databases using the search terms ‘genome-wide screen’ followed procedurally by each ‘<virus species>’ from the human-infecting viruses list; this search was performed on 10–11 April 2024.

### Data eligibility

Publications were included based on the following criteria to avoid bias:

1. Peer-reviewed, full-text manuscript was available to confirm eligibility.
2. Screen was genome-wide and not a subset of genes.
3. Screen involved analysis of gene loss-of-function. Gain-of-function screens were excluded.
4. Studies that used pseudotyped or gene-deleted viruses, such as replication-incompetent viruses or virus-like particles, were excluded. Reporter viruses whereby a reporter gene (e.g. fluorescent protein or luciferase) was inserted into the genome were included if they were fully replication competent.
5. Screen was performed in vertebrate cells.
6. Cells were not treated with any additional agents or drugs.
7. Data was available for, at a minimum, genes classified as significant based on *p*-value, false discovery rate or similar. Screens presenting only arbitrarily defined ‘top hits’ were excluded.
8. Data was able to be ranked by *p*-values, scores, fold change or similar.
9. Data included at least 30 significant genes.

Metadata for included screens is described in Table S2.

### Data processing and ranking

To enable comparison, gene names from each dataset were unified using HUGO Gene Nomenclature Committee (HGNC) [31] to convert all gene identifiers to HGNC-approved names, symbols and IDs. Any gene names not found using HGNC were searched for synonyms using gene (National Centre for Biotechnology Information). Data from non-human species were first converted to human orthologues using Gene and Ortholog Location Finder [32,33] for *Mus musculus* genes and UniProt ID Mapping tool [34] for *Chlorocebus sabeus* genes. Duplicate genes and non-protein-coding genes (microRNAs, pseudogenes, non-coding RNAs, etc.) were removed. Where more than one dataset was available for a particular virus, to allow for integration of diverse datasets, we performed robust rank aggregation (RRA) using GeneRaMeN [35]. This analysis takes multiple ranked datasets and integrates them into a single ranked consensus dataset whereby genes that appear more frequently and at higher rankings across the input datasets will be ranked higher in the consensus list. In this way, we generated a ranked consensus list of host factors for each virus, virus family and across all families. Ranked input datasets, as well as family and pan-family consensus lists, are available in Data S1.

### Pathway analysis and gene clustering

To identify related proteins and cellular pathways important to infection, protein–protein interaction networks (PPINs) were generated with STRING [36] using the top 50 ranked host factors from the virus family and pan-family consensus lists. This analysis generates clusters of proteins with predicted associations based on factors, such as co-expression, experimental evidence, database and text mining, and genomic factors to generate an interaction confidence score. Only protein associations with at least a ‘high’ confidence score (> 0.7) were included here.

## Results

### Literature search

**Fig. 1.**
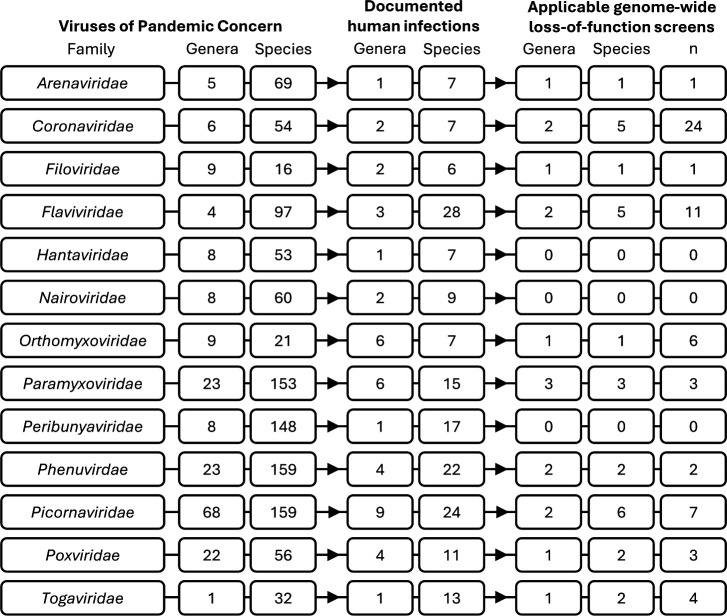
Phylogenetic overview of virus families of pandemic concern and applicable functional genomic screens. Number of genera and species belonging to virus families of pandemics concern (ICTV Master Species List #39 [29]), including those with documented human infections (Virus–Host Database [30]) and genome-wide loss-of-function screens that met the criteria.

**Fig. 2.**
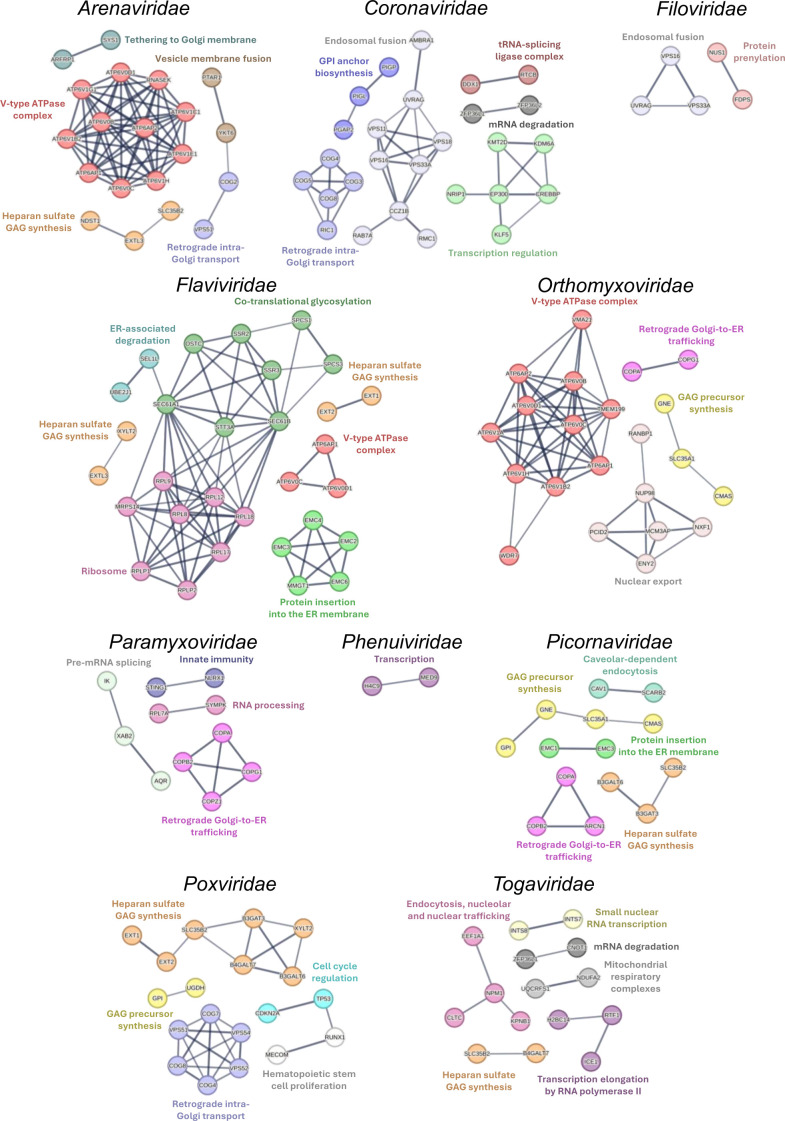
PPIN of top pandemic virus family host factors. The top 50 ranked host factors from each family dataset were used to create PPINs using STRING [36]. Nodes belonging to the same PPIN share the same colour, which is conserved between families. Lines between nodes denote the interaction score confidence as calculated by STRING (thin line, high confidence; thick line, highest confidence). Cellular processes associated with each cluster are also shown.

A total of 62 datasets covering 10 out of 13 families were identified (Fig. 1), no screens that met the criteria were available for viruses belonging to the *Hantaviridae*, *Nairoviridae* or *Peribunyaviridae* families. PPIN analysis of the top 50 ranked host factors for each family is shown in Fig. 2.

### Arenaviridae

Only a single dataset was available for arenaviruses, investigating lymphocytic choriomeningitis virus (LCMV) [37]. The largest cluster included multiple components of the V-type ATPase proton transport complex (V-ATPase) and accessory proteins (*ATP6VB2*, *ATP6V0C*, *ATP6V0B*, *ATP6V1G1*, *ATP6AP1*, *ATP6V0D1*, *ATP6V1C1*, *ATP6V1E1*, *ATP6V1H*, *ATP6AP2* and *RNASEK*) [38,39]. Smaller clusters included host factors involved in heparan sulphate (HS) glycosaminoglycan (GAG) synthesis (*EXTL3*, *NDST1* and *SLC35B2*), tethering to the Golgi membrane (*ARFRP1*, *SYS1*), retrograde intra-Golgi transport (*COG2*, *VPS51*) and vesicle membrane fusion (*YKT6*, *PTAR1*) [40,48].

### Coronaviridae

Coronaviruses had the highest number of datasets, including 14 for SARS-CoV-2 [9,27, 49,56], 2 for Middle East respiratory syndrome coronavirus [56,57], 4 for human coronavirus (HCoV)−229E [9,49, 57, 58], 3 for HCoV-OC43 [9,49, 51], and a single dataset for HCoV-NL63 [49]. PPIN clusters contained host factors involved in endosomal fusion (*VPS11*, *VPS16*, *VPS18*, *VPS33A*, *CCZ1B*, *RAB7A*, *RMC1*, *UVRAG* and *AMBRA1*) [45,59, 60]. Other clusters were related to transcription regulation and histone modification (*KMT2D*, *KDM61*, *CREBBP*, *EP300*, *NRIP1* and *KLF5*), retrograde intra-Golgi vesicular transport (*COG3*, *COG4*, *COG5*, *COG8* and *RIC1*) [46,61,66], glycosylphosphatidylinositol (GPI)-anchor biosynthesis (*PIGL*, *PGAP2* and *PIGP*), tRNA-splicing ligase complex (*DDX1*, *RTCB*) and mRNA degradation (*ZFP36L1*, *ZFP36L2*) [67,69].

### Filoviridae

Only a single dataset was available for filoviruses investigating the Zaire Ebola virus (EBOV) [70]. PPIN analysis revealed two clusters involving endosomal fusion (*VPS16*, *VP33A* and *UVRAG*) and protein prenylation (*NUS1*, *FDPS*) [45,59, 60, 71, 72].

### Flaviviridae

Flavivirus had the second highest number of datasets, including three studies for both Zika virus (ZIKV) [73,74] and West Nile virus (WNV) [75,77], 2 for Dengue virus (DENV) [73,78] and HCV [79,80] and a single study for yellow fever virus (YFV) [81]. Clusters of host factors involved in flavivirus infection encompass protein translation and post-translational modification at the endoplasmic reticulum (ER) membrane, including ribosomal proteins (*RPL8*, *RPL9*, *RPL12*, *RPL17*, *RPL18*, *RPLP1*, *RPLP2* and *MRPS14*), co-translational glycosylation (*OSTC*, *SST3A*, *SEC61A1*, *SEC61B*, *SSR2*, *SSR3*, *SPCS1* and *SPCS3*) and the ER-associated degradation pathway (*SEL1L*, *UBE2J1*) [82,84]. Members of the ER-membrane insertase complex (EMC) were also enriched (*EMC2*, *EMC3*, *EMC4*, *EMC6* and *MMGT1*) [85]. Remaining clusters include V-ATPase components (*ATP6V0C*, *ATP6V0D1* and *ATP6AP1*) and HS-GAG synthesis (*EXT1*, *EXT2* and *EXTL3*, *XYLT2*) [38,40, 41].

### Orthomyxoviridae

Six studies investigated host factors related to orthomyxovirus infection, all focussed on influenza A virus (IAV), including H1N1, H5N1 and H7N9 subtypes [86,91]. Members of the V-type ATPase complex, and related proteins, were enriched (*ATP6V0B*, *ATP6V0C*, *ATP6V0D1*, *ATP6V1A*, *ATP6V1B2*, *ATP6V1H*, *ATP6AP1*, *ATP6AP2*, *TMEM199*, *VMA21* and *WDR7*) [38,92, 93]. A secondary cluster contained host factors involved in nuclear export (*ENY2*, *MCM3AP*, *PCID2*, *NXF1*, *NUP98* and *RANBP1*) [94,99]. Remaining clusters include host factors relevant to GAG precursor synthesis (*GNE*, *SLC35A1* and *CMAS*) and retrograde Golgi-to-ER trafficking (*COPA*, *COPG1*) [100,101].

### Paramyxoviridae

Single datasets were available for three paramyxoviruses, Hendra virus [102], measles virus [8] and mumps virus [8]. PPIN analysis revealed an importance for retrograde Golgi-to-ER trafficking components (*COPA*, *COPB2*, *COPG1* and *COPZ1*), pre-mRNA splicing (*XAB2*, *AQR* and *IK*), RNA processing (*RPL7A*, *SYMPK*) and innate immunity (*STING*, *NLRX1*) [101,103,110].

### Phenuiviridae

Two datasets were available for phenuiviruses, one each for Heartland virus [111] and Rift Valley fever virus [112]. Only a single cluster containing two host factors was enriched, with functions in transcription (*H4C9*, *MED9*) [113,114].

### Picornaviridae

Datasets were available for several species. This includes two for enterovirus 71 (EV71) [115,116] and single datasets for enterovirus D68 (EVD68) [117], hepatitis A virus [118], poliovirus [119], rhinovirus B [120] and rhinovirus C [117]. Host factor clusters included GAG precursor synthesis (*GPI*, *GNE*, *SLC35A1* and *CMAS*), HS-GAG synthesis (*B3GAT3*, *B3GALT6* and *SLC35B2*), retrograde Golgi-to-ER trafficking (*COPA*, *COPB2* and *ARCN1*) and EMC complex (*EMC1*, *EMC3*) [40,41, 85, 100, 101]. Unique to this family were host factors related to caveolar-dependent endocytosis (*CAV1*, *SCARB2*) [121,122].

### Poxviridae

Investigation of poxvirus host factors uncovered two datasets investigating Mpox virus (MPXV) [123] and a single dataset for vaccinia virus (VACV) [124]. Two clusters containing several host factors participating in HS-GAG synthesis (*EXT1*, *EXT2*, *SLC35B2*, *B3GAT3*, *B3GALT6*, *B4GALT7* and *XYLT2*) or intra-Golgi retrograde transport (*COG4*, *COG7*, *COG8*, *VPS51*, *VPS52* and *VPS54*) were identified [40,41, 101]. Host factors of the remaining clusters are involved in GAG precursor biogenesis (*GPI*, *UGDH*), hematopoietic stem cell proliferation (*MECOM*, *RUNX1*) and cell cycle regulation (*TP53*, *CDKN2A*) [100,125,127].

### Togaviridae

Four screens were available for togaviruses, two each for chikungunya virus (CHIKV) [128,129] and Sindbis virus (SINV) [130,131]. The cluster with the largest number of host factors involved diverse functions in endocytosis and nucleolar/nuclear trafficking (*NPM1*, *KPNB1*, *CLTC* and *EEF1A1*) [132,135]. Several clusters featured host factors involved in RNA biogenesis, including transcription elongation by RNA polymerase II (*RTF1*, *ICE1* and *H2BC14*), mRNA degradation (*ZFP36L1*, *ZFP36L2*) and small nuclear RNA synthesis (*INTS7*, *INTS8*) [69,136,142]. Remaining gene clusters were involved in HS-GAG synthesis (*SLC35B2*, *B4GALT7*) and mitochondrial respiratory complexes (*NDUFA2*, *UQCRFS1*) [40,41, 143].

### Pan-family host factors

**Fig. 3.**
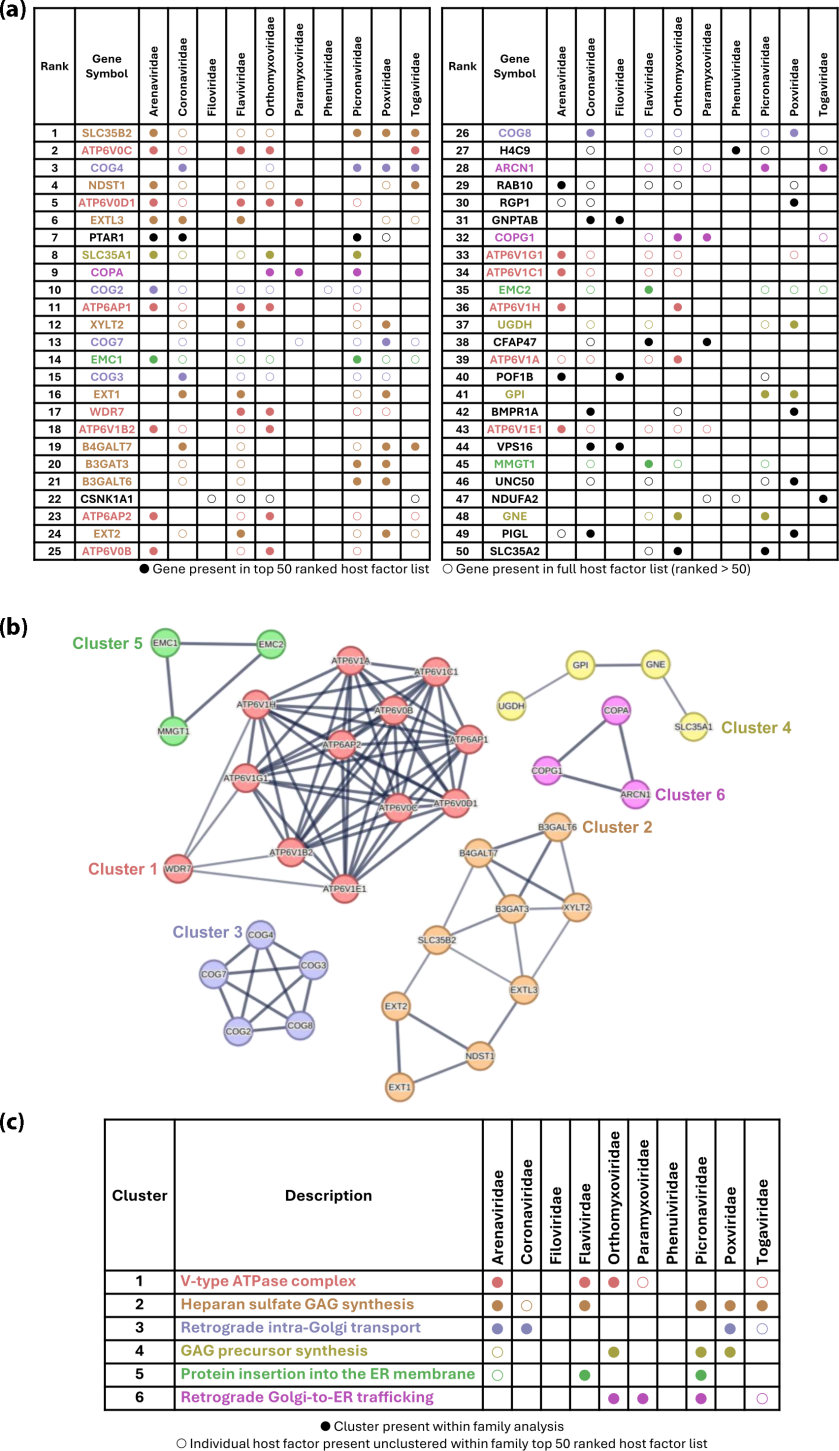
Host factors involved in infection by multiple virus families. (**a**) Top 50 pan-family ranked host factors. Corresponding host factors found at the family analysis stage are denoted by a dot, with a filled dot indicating presence in the top 50 ranked genes for that family and an open dot indicating presence amongst the full gene list (ranked >50). Genes are colour coded corresponding to the PPINs shown in (b). (**b**) PPIN clusters based on the host factors shown in (a) using STRING [36]. Nodes belonging to the same PPIN share the same colour. Lines between nodes denote the interaction score confidence as calculated by STRING (thin line, high confidence; thick line, highest confidence). (**c**) Cellular processes associated with each cluster in (b) are shown. Corresponding clusters found at the family analysis stage are denoted by filled dots and empty dots denote the presence of one or more unclustered individual genes belonging to that cluster in the top 50 ranked host factor lists of that family.

Using the consensus host factor lists for each family, further RRA analysis was performed to generate a consensus list of pan-family host factors. The top 50 ranked pan-family host factors (Fig. 3a) were subject to PPIN analysis (Fig. 3b). These clusters contained host factors involved in the V-ATPase (Cluster 1), HS-GAG synthesis (Cluster 2), retrograde intra-Golgi transport (Cluster 3), GAG precursor synthesis (Cluster 4), protein insertion into the ER membrane (Cluster 5) and retrograde Golgi-to-ER trafficking (Cluster 6). Conserved clusters of host factors were observed in at least two individual families (Fig. 3c), indicating broad relevance of these cellular pathways in the replication cycle of diverse viruses. No conserved clusters and very few conserved host factors were identified for filoviruses and phenuiviruses.

**Fig. 4.**
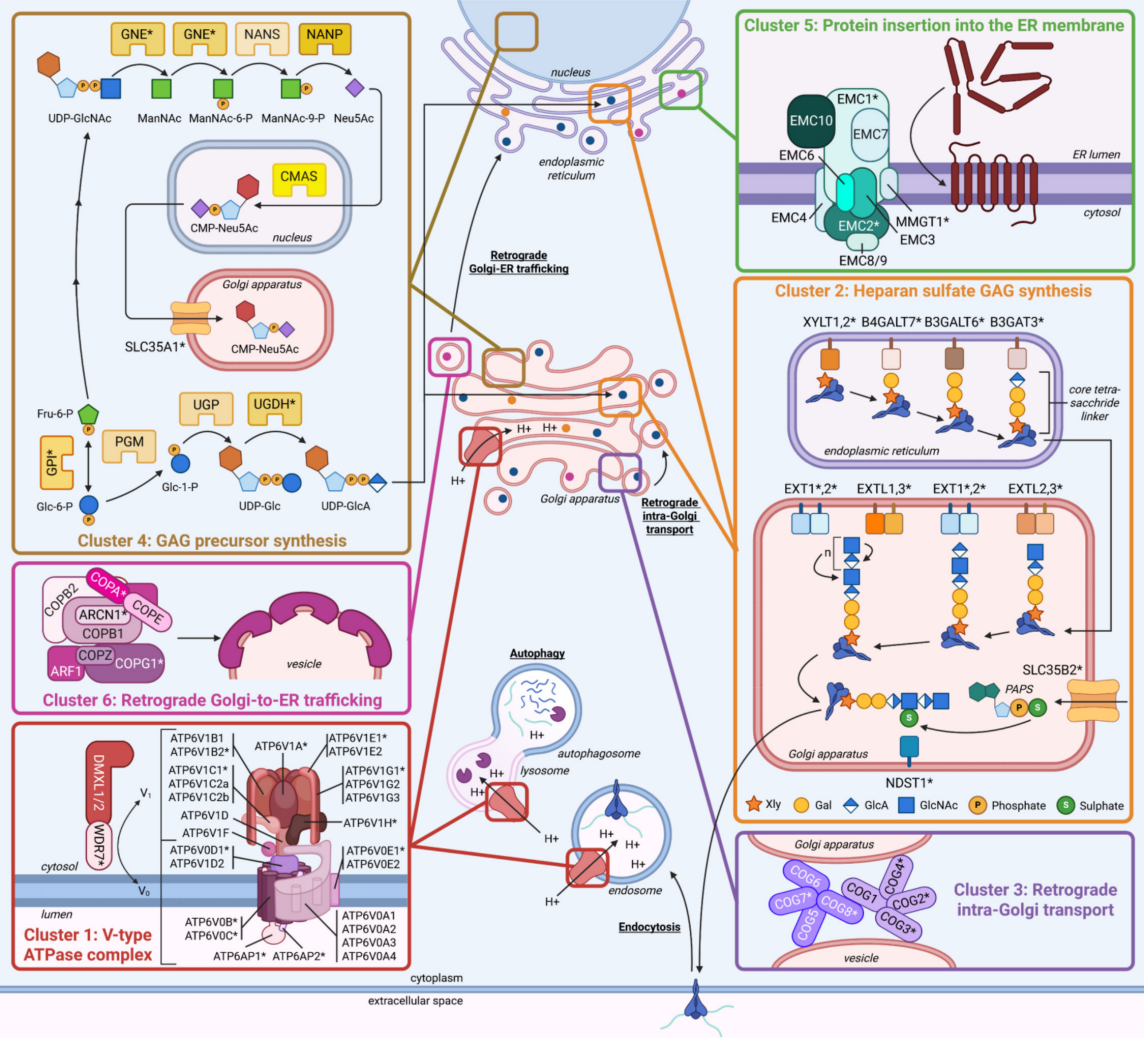
Pan-family virus–host factors have diverse cellular functions. A diagram of the cellular functions of pan-family PPIN clusters is shown in Fig. 3(b). Host factors shown in Fig. 3(a) are denoted by an asterisk (*). Cluster 1 depicts the V-ATPase, composed of membrane-bound V_0_ and soluble V_1_ sub-complexes [38]. The complex transports protons across membranes in an ATP-dependent manner to induce acidification of certain cellular compartments such as vesicles and the Golgi apparatus, contributing to processes such as endocytosis, autophagy and post-translational modification [38]. The Rabconnectin-3 complex (WDR7 and DMXL1/2) facilitates the assembly of V_1_ onto V_0_ [92]. Cluster 2 depicts HS-GAG synthesis. A tetrasaccharide linker is synthesized in the ER through sequential addition of Xyl-Gal-Gal-GlcA by the indicated enzymes [40]. Heparan chains are appended within the Golgi apparatus, beginning with the addition of GlcNAc by the EXTL2,3 dimer and GlcA by EXT1,2 [40]. Chain elongation involving the addition of repeating GlcA and GlcNAc units is performed by EXT1,2, while EXTL1,3 can only catalyse the addition of GlcNAc to GlcA [40,41]. Sulfation of heparan is performed by NDST1 using the sulphate donor PAPS, which is imported into the Golgi by SLC35B2 [40,41]. HS-GAGs are commonly found on the cell surface [145]. Cluster 3 depicts the COG complex that forms an interaction hub between the Golgi membrane and various factors on vesicle membranes, forming a tethering complex, to promote fusion or trafficking; this is critical for retrograde intra-Golgi transport [46]. Cluster 4 depicts the synthesis of precursor molecules used for the synthesis of GAGs. GPI interconverts Glc-6-P and Fru-6-P that act as starting molecules for the synthesis of different GAG precursors [251]. Glc-6-P is procedurally converted to UDP-GlcA by PGM, UGP and UGDH and is used as a GlcA donor within the ER and Golgi apparatus [251]. Fru-6-P undergoes several enzymatic steps to be converted into UDP-GlcNAc [100], whereby the enzymes GNE, NANS and NAP convert this molecule into Neu5Ac [100]. The terminal step occurs within the nucleus whereby CMAS catalyses the addition of CMP, forming CMP-Neu5Ac [100]. This molecule is then imported into the nucleus by the transport SLC35A1 and serves as a key sialic acid donor [100]. Cluster 5 depicts the EMC, a multi-subunit transmembrane insertase complex that mediates the co-translational insertion of proteins into the ER membrane [85]. Cluster 6 depicts the COPI coatomer that primarily mediates retrograde Golgi-ER vesicular transport by forming a ‘coat’ around vesicles to direct interactions with tethering factors [101].

The cluster containing the most host factors was linked to components of the V-ATPase (Cluster 1), a membrane-embedded multi-subunit rotary complex that translocates protons across membranes in an ATP-dependent manner (Fig. 4) [38]. Conserved clusters containing similar host factors were observed for arenaviruses, flaviviruses and orthomyxoviruses, while unclustered host factors were found for paramyxovirus and togavirus. The primary role of the V-ATPase is to facilitate the acidification of cellular compartments, including vesicles and the Golgi apparatus [38]. Within endosomes, the acidic pH contributes to pH-dependent sorting and/or release of cargoes, while low pH in lysosomes is necessary for the activity of acidic degradative enzymes involved in autophagy [38,144]. Glycosylation of proteins within the Golgi (Cluster 2) and retrograde Golgi-to-ER (Cluster 6) and intra-Golgi (Cluster 3) transport are also pH-sensitive processes that are maintained by the V-ATPase [144]. Alongside the V-ATPase components was clustered WD-repeat domain 7 (WDR7) that is a subunit of the Rabconnectin-3 complex [92]. This complex facilitates the assembly of the soluble V_1_ and membrane-bound V_0_ subcomplexes [92].

Cluster 2 includes genes involved in GAG synthesis, specifically HS. Related genes were found in clusters for arenaviruses, flavivirus, picornaviruses, poxviruses and togaviruses, while unclustered genes were observed for coronaviruses. HS-GAG synthesis takes place across the ER and the Golgi. Within the ER, the core tetrasaccharide linker (Xyl-Gal-Gal-GlcA) common to most GAGs is progressively conjugated to proteins by xylotransferase 1 or 2 (XYLT1, XYLT2), beta-1,4-galactosyltransferase 7 (B4GALT7), beta-1,3-galactosyltransferase 6 (B3GALT6) and beta-1,3-*N*-acetylgalactosaminyltransferase 1 (B3GALNT1/B3GAT3) [40]. Proteins possessing this core tetrasaccharide are then transported to the Golgi where more complex glycosylation occurs [40]. In the case of HS, this involves heterodimeric complexes of exostosin (EXT) and exostosin-like (EXTL) enzymes, EXTL2/3, EXT1/2 and EXTL1/3 [40]. Solute carrier family 35 member B2 (SLC35B2) is a Golgi membrane transporter responsible for the import of 3′-phosphoadenosine 5′-phosphosulfate (PAPS), a sulphate donor used in HS-GAG synthesis by *N*-deacetylase and N-sulfotransferase 1 (NDST1) [40,41]. HS-GAGs are commonly found on the cell surface and extracellular matrix where they carry out a variety of biological functions, including as coreceptors and facilitating endocytosis [145].

Cluster 3 includes multiple members of the Conserved Oligomeric Golgi (COG) complex that form a tethering complex between the Golgi membrane and various factors located on the membrane of vesicles [46]. This promotes either membrane fusion or retrograde trafficking of vesicles within the Golgi (from the trans to cis end) [46]. Analysis of arenavirus, coronavirus, poxvirus and togavirus host factors indicates the importance of the COG complex to infection. Notably, disruptions to one or more COG proteins have been observed to lead to defects in glycosylation and/or Golgi fragmentation [46], highlighting a link between the COG complex and HS-GAG synthesis (Cluster 2).

Host factors in Cluster 4 are involved in the synthesis of precursor molecules used in the generation of GAGs, including HS (Cluster 2). Two enzymatic pathways are linked by glucose-6-isomerase (GPI), which interconverts glucose-6-phosphate (Glc-6-P) and fructose-6-phosphate (Fru-6-P) [146]. Glc-6-P is used for the synthesis of uridine diphosphate-glucuronic acid (UDP-GlcA), a process involving UDP-glucose-6-dehydrogenase (UGDH) at the terminal step [146]. UDP-GlcA is used in the synthesis of HS and other GAGs [146]. Fru-6-P is instead used for the synthesis of cytosine monophosphate-N-acetylneuraminic acid (CMP-Neu5Ac), the most common sialic acid, involving the activity of bifunctional UDP-*N*-acetylglucosamine 2-epimerase/*N*-acetylmannosamine kinase (GNE) [100]. Solute carrier family 35 member A1 (SLC35A1) is involved in the transport of CMP-Neu5Ac into the Golgi [100]. Poxvirus host factor clusters included GPI and UGDH, indicating importance to HS-GAG synthesis, and consistent with a corresponding HS-GAG cluster for this family. In contrast, an orthomyxovirus cluster included host factors involved in sialic synthesis, including SLC35A1, GNE and cytidine monophosphate *N*-acetylneuraminic acid synthetase (CMAS). The related cluster for picornaviruses contained host factors from both pathways, GPI, GNE, SLC35A1 and CMAS.

Cluster 5 is composed of subunits of the EMC, a multi-subunit transmembrane complex that functions as an insertase, mediating the co-translational insertion of proteins into the ER membrane [85]. Conserved clusters were identified for flaviviruses and picornaviruses.

Cluster 6 consists of components of the COPI coatomer [101]. The heteroheptameric COPI coatomer forms a ‘coat’ around vesicles destined for transport to the ER-intermediate complex (ERGIC)/ER and facilitates interactions with tethering factors to mediate transport [101]. Commonly, these vesicles contain ER-resident proteins returning to the ER following post-translational modification or Golgi-resident proteins intended for recycling [101]. This cluster was relevant to orthomyxoviruses, picornaviruses and the only conserved cluster for paramyxoviruses.

### Existing inhibitors of host factors required for virus replication

A review of literature has identified small-molecule inhibitors for members of Clusters 1, 2 and 6, as well as some unclustered proteins (Table 1). There has been interest in V-ATPase (Cluster 1) inhibitors in the treatment of cancer and osteoporosis [146,147]. V-ATPases contribute to the metastasis of cancers by promoting the activity of acidic proteases that degrade the extracellular matrix, while also protecting tumour cells from apoptosis through regulation of cellular pH [146]. V-ATPases are also abundantly expressed in osteoclasts and contribute to osteoporosis through their regulation of extracellular pH to promote bone reabsorption [147]. Several experimental drugs have been developed that target the V-ATPase, but none have yet progressed to clinical trials for the treatment of these diseases. One drug, enoxacin, is a fluoroquinolone antibiotic that was approved as an anti-infective but has been shown to have off-target effects in inhibiting the actin binding domain of ATP6V1B2 [147]. Approval for this drug has since been withdrawn due to adverse effects, though these have not been attributed to its effects on the V-ATPase [148]. V-ATPase inhibitors have been found to inhibit infection by various viruses, including SINV, IAV, EBOV, SARS-CoV-2, Japanese encephalitis virus and ZIKV [149,157].

**Table 1.** Small-molecule inhibitors targeting pan-family virus–host factors identified in this analysis

	Gene ID	Name	Status	Ref
Cluster 1	ATP6V0C	Concanamycin	Experimental	[252]
		Bafilomycin	Experimental	[253]
		Salicylihalamide	Experimental	[254]
		Apicularens	Experimental	[255]
		Lobatamides	Experimental	[256]
		Oximidines	Experimental	[257]
		Indolyls	Experimental	[258]
		Archazolid	Experimental	[255]
	ATP6V1B2	Enoxacin	Withdrawn	[259]
		KM91104	Experimental	[260]
	V-ATPase complex	NiK12192	Experimental	[261]
		SB242784	Experimental	[262]
		FR202126	Experimental	[242]
		3-Bromopyruvate	Experimental	[263]
		Tributyltin chloride	Experimental	[264]
		FR177995	Experimental	[243]
		FR167356	Experimental	[265]
		Diphyllin	Experimental	[266]
		Iejimalides	Experimental	[267]
Cluster 2	NDST1	Peptides	Experimental	[160]
	B4GALT7	*β*-d-Xylopyranoside analogues	Experimental	[162]
	B3GAT3	TMLB-C16	Experimental	[161]
Cluster 6	COPI complex (*via* GBF1)	Brefeldin A	Experimental	[163,164]
Unclustered	CSNK1A1	D4476	Experimental	[268]
		CHEMBL2203564	Experimental	[269]
		IC261	Experimental	[270]
		CHEMBL2203552	Experimental	[269]
		CHEMBL2203555	Experimental	[269]
	BMPR1A	Dorsomorphin	Experimental	[168]
		CHEMBL2385579	Experimental	[173]
		CHEMBL2385582	Experimental	[173]
		CHEMBL2385596	Experimental	[173]
		CHEMBL513147	Experimental	[172]
		LDN-214117	Experimental	[171]
		CHEMBL3818173	Experimental	[171]
		ML347	Experimental	[173]
		CHEMBL511563	Experimental	[173]
		CHEMBL4280599	Experimental	[172]
	Mitochondrial complex I (includes NDUFA2)	Metformin	Clinical	[174]

Experimental drugs have also been developed targeting enzymes involved in GAG synthesis in the context of cancer, whereby they contribute to multiple processes relevant to tumour cell proliferation, invasion and metastasis [158,159]. Peptide-based inhibitors have been developed targeting NDST1, disrupting sulfation of GAGs [160]. Others are more general inhibitors of GAG synthesis by targeting enzymes involved in the synthesis of the core tetrasaccharide, including B4GALT7 and B3GAT3 [161,162]. Brefeldin A (BFA) is used as a research tool to study protein transport and Golgi function by inhibiting the formation of the COPI coatomer [163]. The mechanism of action is postulated to be through inhibition of Golgi-specific BFA-resistance guanine nucleotide exchange factor 1 (GBF1), preventing conversion of ADP-ribosylation factor 1 (ARF1)-GDP to ARF1-GTP to enable its recruitment of COPI [164].

Three unclustered host factors have also been of interest for therapeutic development, *CSNK1A1*, *BMPR1A* and *NDUFA2*. Casein kinase 1*α* (CK1*α*), encoded by *CSNK1A1*, has a variety of cellular roles, including as a regulator of Wnt/*β*-catenin, NFκB and p53 signalling pathways [165]. These provide CK1*α* relevance in cancer, inflammation and neurodegenerative disorders [165]. Bone morphogenetic protein receptor type 1A (BMPR1A), also referred to as activin-like kinase 3 (ALK3), is involved in bone and cartilage morphogenesis, embryogenesis, homeostasis and inflammation [166,169]. The related protein, ALK2, is of interest for drug development to treat the rare condition, fibrodysplasia ossificans progressiva, that results in soft tissue ossification [170]. Drugs designed to target ALK2 often also target ALK3 [171,173]. The only clinically approved drug is metformin, used in the treatment of type 2 diabetes mellitus [174]. Part of its mechanism of action is through inhibition of the first complex in the electron transport chain, mitochondrial complex I, which includes NADH:ubiquinone oxidoreductase subunit A2 (NDUFA2) [174]. The specific mechanism of action of metformin on complex I has not been fully elucidated, though studies with related compounds have shown multiple binding sites that do not involve a specific interaction with NDUFA2 [175].

## Discussion

**Fig. 5.**
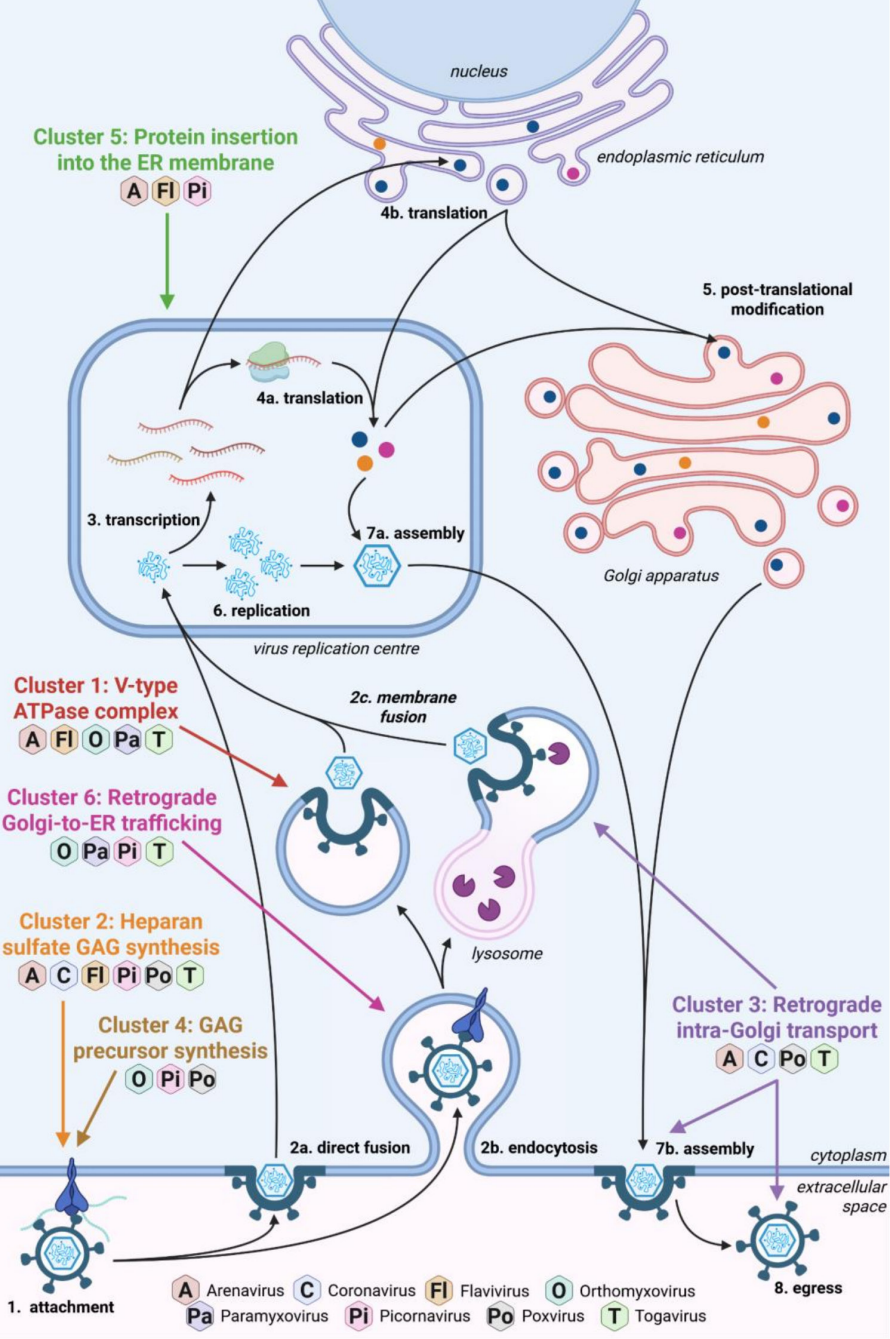
Potential roles for the pan-family host factors in the virus life cycle. Diagram of a generic virus life cycle. (1) Viruses use attachment factors and/or receptors to bind to the cell surface, facilitating entry either by (2a) direct fusion or (2b) endocytosis and subsequent (2c) membrane fusion to release the virion contents. Viruses typically establish a replication centre within the infected cell where (3) transcription occurs. Translation may occur within the replication complex using (4a) free ribosomes or (4b) mRNA is transported to ER for translation by ER-resident ribosomes. Viral surface proteins commonly undergo (5) post-translational modification in the Golgi apparatus and are often trafficked to the plasma membrane. Assembly of virions may occur within (7a) replication centres and/or at the (7b) plasma membrane where they acquire the viral envelope and surface proteins and (8) bud from the cell. Stages where host factors from the indicated clusters (from Figs 4 and 5) are proposed to facilitate the virus life cycle are indicated. The virus families that require these host factors based on the meta-analysis are also shown.

The clusters identified in this study are predominantly associated with the early stages of the virus life cycle but may also facilitate later stages of infection (Fig. 5). Foremost, host factors in Cluster 2 (HS-GAG synthesis) and Cluster 4 (GAG precursor synthesis) are involved in GAG synthesis. Cell surface GAGs are commonly proposed as viral attachment factors or entry receptors, the best known being orthomyxoviruses utilizing sialic acid for cell entry [176]. In addition to orthomyxoviruses, this meta-analysis also identified roles for sialic acid (Cluster 4) in infection of picornaviruses. This is reflected in the literature whereby picornaviruses Coxsackie virus A24, EVD68 and EV70 have been shown to have a dependence on genes involved in sialic acid synthesis for infection and, more specifically, sialylated GAGs act as attachment factors and/or cell entry receptors for these viruses [177,179]. A reliance on HS-GAGs for infection was also identified (Cluster 2), namely, for arenaviruses, flaviviruses, picornaviruses, poxviruses and togaviruses. HS-GAGs have been shown to act as cell receptors for viruses belonging to these families [180,188]. Several genes belonging to Clusters 2 and 4 have been validated as virus–host factors by targeted knockdown/overexpression, including *SLC35B2* (EV71, Saffold virus), *B3GAT3* (EV71), *NDST1* (CHIKV), *EXTL3* [vesicular stomatitis virus (VSV) pseudotyped with LCMV glycoprotein], *EXT1* (ZIKV) and *SLC35A1* (H3N2 IAV) [116,187, 189,192]. Intriguingly, reduced expression of *SLC35B2* in mice increased susceptibility to VACV, proposed to be associated with roles in innate immunity [190].

Following cell attachment and entry, viruses must then release the contents of their virions into the cell. This may involve host factors that are found in Cluster 1 (V-ATPase complex), Cluster 3 (retrograde intra-Golgi transport) and potentially also Cluster 6 (retrograde Golgi-to-ER trafficking). Viruses commonly enter cells through endocytic pathways, often requiring either low pH or the activity of acidic proteases to promote virus fusion and uncoating. This low pH environment is facilitated by the V-ATPase (Cluster 1) [38]. Of the viruses examined here, all have been observed to enter cells *via* endosomal pathways, except for paramyxoviruses [176,193,201]. Indeed, several V-ATPase inhibitors (Table 1) have been shown to be efficacious against these viruses both *in vitro* and *in vivo* [152,156, 202,209]. Paramyxoviruses instead enter cells *via* pH-independent routes, primarily through direct fusion with the cellular membrane, though some endocytic events may also contribute to entry of some family members, namely, henipaviruses [210,211]. This meta-analysis indicated the importance of the V-ATPase in infection by arenaviruses, flaviviruses and orthomyxoviruses, though V-ATPase components were also observed in the host factor lists for coronaviruses, paramyxoviruses, picornaviruses, poxviruses and togaviruses, but they either fell outside the top 50 host factors and/or were insufficient in number to form a distinct cluster. This may reflect observations of multiple modes of cell entry used by these viruses. Coronaviruses and poxviruses additionally utilize direct fusion, while picornaviruses may also enter cells through the caveosome, an alternative pH-independent endocytic pathway [193,196]. As paramyxoviruses’ cell entry is pH-independent, the role of the V-ATPase in infection by these viruses may involve its additional roles in protein trafficking and/or glycosylation (discussed above) [144]. Despite the apparent necessity for pH-dependent cell entry for filoviruses and phenuiviruses, the V-ATPase genes were completely absent from their list of host factors, perhaps indicating alternative pH-independent methods of entry that are yet to be described.

Based on the functions of COPI in retrograde Golgi-to-ER trafficking (Cluster 6), it could suggest roles in assembly for orthomyxoviruses, paramyxoviruses and picornaviruses. COPI vesicles could be hypothesized to shuttle viral glycoproteins from the Golgi to sites of replication. However, picornaviruses are non-enveloped viruses and have not been shown to encode glycoproteins. Orthomyxoviruses and paramyxoviruses assemble at the plasma membrane [212,213]; therefore, COPI vesicles are unlikely to be utilized for this purpose. Intriguingly, studies have proposed roles for COPI in virus entry, whereby loss of COPI disrupted IAV infection at the stage of early-to-late endosome trafficking [214,215]. Although COPI has been observed to associate with early endosomes and early models proposed roles for COPI in endosomal trafficking, its impact on endocytosis has been difficult to determine due to the general perturbation of the Golgi caused by COPI deficiency that results in defects in the trafficking of endosomal factors [216]. Considering COPI components were not more broadly observed across virus families in this meta-analysis, this general defect is unlikely to explain their importance to virus infection. Studies in yeast have attempted to resolve the role of COPI in endosomes, revealing that it is involved in cargo sorting by mediating the return of exocytic SNAP-related proteins (SNAREs) from the plasma membrane back to the Golgi; SNAREs are involved in targeting vesicles to different membranes and mediate their fusion [216]. Based on this finding, it could be instead hypothesized that viruses may utilize this pathway for cell entry. Indeed, a similar entry process has been proposed for Hepatitis B virus, a hepadnavirus, which utilizes the retrograde Golgi trafficking pathway for transport from the early endosome to the nucleus in a COPI-dependent manner, potentially to circumvent lysosomal degradation and immune detection [217]. As IAV also replicates within the nucleus, it is intriguing to speculate that it may use an analogous method of cell entry. BFA has, however, been shown to interfere with the intracellular processing of IAV haemagglutinin and neuraminidase [218,220]. COPI has also been proposed to be variously involved in the formation of replication centres by picornaviruses based on their replication being sensitive to BFA, a COPI inhibitor [221,222]. This is further supported by localization of COPI components to picornavirus replication centres and interactions between viral proteins with COPI and COPI-related host factors [223,224]. As for paramyxoviruses, links with COPI have not been shown, but considering its prominence amongst paramyxovirus clusters, further investigation is warranted.

Cluster 3 (retrograde intra-Golgi transport) encompassed components of the COG complex, and the meta-analysis identified roles for this complex in the life cycles of arenaviruses, coronaviruses and poxviruses. In the literature, it has been observed that the entry, fusion and egress of VACV and MPXV are impeded in COG deficient cells [225]. The mechanism is speculated to be indirect through depletion of host factors necessary for these processes and likely relates to the role of the COG complex in protein glycosylation [225]. Roles for COG proteins in arenaviruses infection have also been proposed, with loss of COG proteins impacting entry of a VSV pseudotype with the glycoprotein of the arenavirus, Lujo virus (LUJV) [226]. This could be due to the contribution of COG proteins to the glycosylation of *α*-dystroglycan, the entry receptor of Old World arenaviruses that includes LUJV; this has not been studied in the context of infection, however [227,228]. For coronaviruses, knockout of COG3 has been observed to decrease infection by HCoVs OC43, NL63 and 229E and SARS-CoV-2 [49].

The meta-analysis identified the importance of the EMC (Cluster 5: protein insertion into the ER membrane) in the life cycles of flaviviruses and picornaviruses. Flaviviruses replicate at the ER and insert a viral polyprotein into the ER membrane, highlighting a potential role for the EMC [229,231]. Indeed, aberrant insertion and degradation of the polyprotein of DENV and ZIKV has been observed in EMC-deficient cells and an interaction of the viral NS4B protein and the EMC has been shown [232,236]. An additional role for subunits of the EMC in flavivirus entry has been proposed whereby depletion of EMC4 impeded membrane fusion and genome release of DENV [232,237]. EMC4 and EMC7 have been suggested to have moonlighting function as tethering factors between the Golgi and vesicles, indicating roles in cellular trafficking [238]. Indeed, EMC4 was shown to be involved in the trafficking of phosphatidylserine to endosomes, which was important for virus membrane fusion [237]. Further studies showed roles for EMC4 in ZIKV and YFV infection, but not WNV [232]. Interestingly, WNV is transmitted by *Culex* mosquitoes, while DENV, ZIKV and YFV are transmitted by *Aedes* mosquitoes, revealing a relationship between vector usage and host factor utilization in humans [232]. In the context of infection by picornaviruses, roles for the EMC have not been directly investigated. However, many picornaviruses form replication complexes derived from ER membranes [239,241], highlighting a potential case for the EMC complex contributing to the virus life cycle. Another potential contribution of the EMC to the virus life cycle is protein glycosylation, whereby the EMC cooperates with the Sec61 translocon to mediate cotranslational import of nascent proteins produced by cytosolic ribosomes into the ER and couples this process with glycosylation enzymes [83,85].

A main hesitancy for the use of HDAs is the potential for toxicity [10,11]. Several studies have investigated the effects of therapeutics targeting the pan-family host factors identified here using mouse models. V-ATPase inhibitors have been investigated in the context of cancer metastasis and arthritis [242,244]. These studies did not report any adverse health effects following daily oral administration of small-molecule V-ATPase inhibitors, FR177995 and FR202126, for 16 and 29 days, respectively [242,243]. Similarly, no toxicity was reported following intraperitoneal administration of a V-ATPase targeting nanobody three times weekly for 3 weeks [242,244]. BFA has also been investigated using xenograft cancer models and infection [245,247]. No toxicity was reported following a single intravenous administration of BFA for up to 28 days [246,247]. Multiple doses of a micelle formation of BFA, increasing pharmacokinetic properties, daily for 14 days also did not result in overt signs of toxicity [245]. In contrast, administration of a single dose of a CMP-Neu5Ac mimetic, CMP-3F-NeuAc, which inhibits the activity of sialyltransferases and the production of sialylated GAGs, intravenously to mice resulted in liver dysfunction, in addition to kidney dysfunction and lethality as early as day 10 post-treatment at higher doses [248]. These studies show that targeting these host factors could be well tolerated but may also result in adverse health outcomes dependent on factors such as the target, dose and efficacy of an inhibitor.

Additional reluctance for HDA development stems from poor translation of host factors identified from *in vitro* studies to an *in vivo* setting. Indeed, a phenomenon has been observed whereby viruses passaged in cell culture show increased affinity for HS, which may account for this widespread importance [145]. Nevertheless, clinical strains of DENV have been shown to interact with HS, while a clinical strain of CHIKV was unable to infect cells lacking GAG expression [180,249]. Natural isolates of Venezuelan and eastern equine encephalitis viruses have also been observed to utilize HS [183,250]. Therefore, an additional validation step is necessary for evaluating potential host factors using clinical or natural virus strains to confirm reliance by circulating strains.

Next steps will involve validating these host factors as pan-viral antiviral targets. *In vivo* infection studies, using either genetic or therapeutic approaches, would affirm these targets as *bona fide* virus–host factors, especially if performed using natural or clinical virus strains. This will lead to a short-list of candidates with which to pursue HDA development. This therapeutic approach would offer many advantages, especially in the context of pandemic preparedness, but requires additional investment to overcome potential shortcomings related to potential toxicity and poor target translation. It is hoped that this meta-analysis will help alleviate the latter concern by deriving high confidence targets from broader scientific literature, and this will encourage future studies to explore how HDAs can contribute to pandemic resilience.

## Supplementary material

10.1099/acmi.0.001167.v3 Uncited Supplementary Material 1.

## Abbreviations

BFA: brefeldin A
CHIKV: chikungunya virus
CK1*α*: casein kinase 1*α*
CMAS: cytidine monophosphate *N*-acetylneuraminic acid synthetase
CMP-Neu5Ac: cytosine monophosphate-*N*-acetylneuraminic acid
COG: Conserved Oligomeric Golgi complex
CRISPR: clustered regularly interspaced short palindromic repeats
DAA: direct-acting antiviral
DENV: dengue virus
EBOV: Ebola virus
EMC: ER-membrane insertase complex
ER: endoplasmic reticulum
EV71: enterovirus 71
EVD68: enterovirus D68
EXT: exostosin
EXTL: exostosin-like
GAG: glycosaminoglycan
GPI: glycosylphosphatidylinositol
HCoV: human coronavirus
HCV: hepatitis C virus
HDA: host-directed antiviral
HGNC: HUGO Gene Nomenclature Committee
HIV-1: human immunodeficiency virus-1
HS: heparan sulphate
IAV: influenza A virus
LCMV: lymphocytic choriomeningitis virus
LUJV: Lujo virus
mAb: monoclonal antibody
MPXV: Mpox virus
NDST1: *N*-deacetylase and *N*-sulfotransferase 1
NDUFA2: NADH:ubiquinone oxidoreductase subunit A2
PAPS: 3′-phosphoadenosine 5′-phosphosulfate
PPIN: protein–protein interaction network
RRA: robust rank aggregation
SARS-CoV-2: severe acute respiratory syndrome coronavirus-2
SINV: Sindbis virus
SLC35A1: solute carrier family 35 member A1
UDP-GlcA: uridine diphosphate-glucuronic acid
UGDH: UDP-glucose-6-dehydrogenase
VACV: vaccinia virus
VSV: vesicular stomatitis virus
WDR7: WD-repeat domain 7
WHO: World Health Organization
WNV: West Nile virus
YFV: yellow fever virus
ZIKV: Zika virus
