# Supplementary material for: Meta-analysis of functional genomics studies reveals conserved cellular pathways required by viruses of pandemic concern
Source: Access Microbiol. 2026 Jun 19;8(6):001167.v3. doi: 10.1099/acmi.0.001167.v3 (PMC13281952; doi:10.1099/acmi.0.001167.v3)
Supplement: Uncited Supplementary Material 1. [file acmi-8-01167-s001.pdf]

Supplementary Table S1. Summary of biology, reservoir, transmission routes, associated disease, case-fatality rates and approved therapeutics of pandemic virus families

Family	Genome	Notable Genera	Notable Species	Suspected Reservoir	Primary Transmission Routes	Disease	CFR (%)	Approved Therapeutics			Refs
								Vaccines	mAbs	DAAs	
Arenaviridae	segmented - and +/- ssRNA	*Mammarenavirus*	LCMV	Rodent	Zoonotic (bites, aerosol excreta)	respiratory, neurological	<1 - 35				(1-7)
			LASV	Rodent	Zoonotic (bites, aerosol excreta) Anthroponotic (bodily fluids)	haemorrhagic	1				
Coronaviridae	+ssRNA	*Alphacoronavirus*	HCoV-229E	Human	Anthroponotic (fomite, respiratory droplets, faecal-oral)	respiratory	<1				(8-18)
			HCoV-NL63	Human	Anthroponotic (fomite, respiratory droplets, faecal-oral)	respiratory	<1				
		Betacoronavirus	HCoV-HKU1	Human	Anthroponotic (fomite, respiratory droplets, faecal-oral)	respiratory	<1				
			HCoV-OC43	Human	Anthroponotic (fomite, respiratory droplets, faecal-oral)	respiratory	<1				
			SARS-CoV	Bat	Anthroponotic (fomite, respiratory droplets, faecal-oral)	respiratory	10				
			MERS-CoV	Bat	Anthroponotic (fomite, respiratory droplets, faecal-oral)	respiratory	35				
			SARS-CoV-2	Bat, human	Anthroponotic (fomite, respiratory droplets, faecal-oral)	respiratory	1	X	X	X	
Filoviridae	-ssRNA	*Orthoebolavirus*	EBOV	Bat	Zoonotic (fomites) Anthroponotic (bodily fluids)	haemorrhagic	72	X	X		(19-26)
			SUDV	Bat	Zoonotic (fomites) Anthroponotic (bodily fluids)	haemorrhagic	53				
			BDBV	Bat	Zoonotic (fomites) Anthroponotic (bodily fluids)	haemorrhagic	33				
		Orthomarburgvirus	MARV	Bat	Zoonotic (fomites) Anthroponotic (bodily fluids)	haemorrhagic	80				
Flaviviridae	+ssRNA	*Orthoflavivirus*	DENV	NHP	Arthropod (mosquito)	febrile, haemorrhagic	<1	X			(27-37)
			YFV	NHP	Arthropod (mosquito)	febrile, haemorrhagic	15	X			
			ZIKV	NHP	Arthropod (mosquito) Anthroponotic (sexual, vertical)	febrile, neurological	<1				
			JEV	Bird, pig	Arthropod (mosquito)	febrile, neurological	15	X			
			WNV	Bird	Arthropod (mosquito)	febrile, neurological	<1				
			TBEV	Small mammals	Arthropod (tick)	febrile, neurological (sequelae)	2 - 30	X			
		Hepacivirus	HCV	Human	Anthroponotic (bodily fluids)	liver, hepatocellular carcinoma, chronic	<1	X		X	
Hantavirus	-ssRNA	*Orthohantavirus*	HTNV	Rodent, shrew, mole	Zoonotic (bites, aerosol excreta)	renal, haemorrhagic, respiratory, cardiac	<1 - 60				(38, 39)
			AMRV	Rodent, shrew, mole	Zoonotic (bites, aerosol excreta)	renal, haemorrhagic, respiratory, cardiac	<1 - 60				
			DOBV	Rodent, shrew, mole	Zoonotic (bites, aerosol excreta)	renal, haemorrhagic, respiratory, cardiac	<1 - 60				
Nairoviridae	-ssRNA	*Orthonairovirus*	CCHFV	Tick	Arthropod (tick)	haemorrhagic	12				(40-45)
		Norwavirus	BJNV	Tick	Arthropod (tick)	febrile	<1				
Orthomyxoviridae	segmented	*Alphainfluenzavirus*	IAV	Human, bird	Anthroponotic (respiratory droplets)	respiratory	<1 - 60	X		X	(46-53)

	-ssRNA	*Betainfluenzavirus*	IBV	Human, bird	Anthroponotic (respiratory droplets)	respiratory	<1				
Paramyxoviridae	-ssRNA	*Henipaviruses*	NIV	Bat	Zoonotic (fomites) Anthroponotic (respiratory droplets, bodily fluids)	respiratory, neurological	38 - 73				(54-60)
			HEV	Bat	Zoonotic (respiratory droplets)	respiratory, neurological	57				
		Morbillivirus	MEV	Human	Anthroponotic (fomites, respiratory droplets)	febrile, respiratory	<1	X			
		Orthorubellavirus	MUV	Human	Anthroponotic (fomites, respiratory droplets)	febrile, parotitis	<1	X			
Peribunyavirus	segmented -ssRNA	*Orthobunyavirus*	OROV	NHP, bird, arthropod	Arthropod (mosquito, midges)	febrile, neurological	<1				(61-65)
			LACV	Rodent, arthropod	Arthropod (mosquito)	febrile, neurological	<1				
			BUNV	Rodent, ruminants, arthropod	Arthropod (mosquito, midges)	febrile, respiratory, haemorrhagic	<1				
Phenuiviridae	segemented - and +/- ssRNA	*Phlebovirus*	RVFV	Arthropod	Arthropod (mosquito)	febrile, ocular, neurological, haemorrhagic	28				(66-74)
			TOSV	Arthropod	Arthropod (sandfly)	neurological	<1				
		Bandavirus	HRTV	Arthropod	Arthropod (tick)	febrile, thrombocytopenia, leukopenia, haemorrhagic	5 - 27				
			SFTSV	Arthropod	Arthropod (tick)	febrile, thrombocytopenia, leukopenia, haemorrhagic	5 - 10				
Picornaviridae	+ssRNA	*Enterovirus*	PV	Human	Anthroponotic (respiratory droplets, faecal-oral)	febrile, paralysis	<1	X			(75-80)
			RV	Human	Anthroponotic (respiratory droplets, faecal-oral)	respiratory, gastrointestinal	<1				
			EV	Human	Anthroponotic (respiratory droplets, faecal-oral)	respiratory, gastrointestinal	<1				
			ECHOV	Human	Anthroponotic (respiratory droplets, faecal-oral)	respiratory, gastrointestinal	<1				
			CV	Human	Anthroponotic (respiratory droplets, faecal-oral)	respiratory, gastrointestinal	<1				
		Parechovirus	SAFV	Human	Anthroponotic (faecal-oral)	respiratory, gastrointestinal, paralysis	<1				
Poxviridae	dsDNA	*Orthopoxvirus*	VACV	Cow	Zoonotic (direct contact)	febrile, skin lesions	<1	X		X	(81-86)
			MPX	Rodent	Zoonotic (direct contact) Anthroponotic (bodily fluids	febrile, skin lesions, haemorrhagic, neurological	<1 - 10	X			
		Parapoxvirus	ORFV	Goat	Zoonotic (direct contact)	febrile, skin lesions	<1				
		Yatapoxvirus	YMTV	NHP	Arthropod (mosquito)	febrile, skin lesions	<1				
			TANV	NHP	Arthropod (mosquito)	febrile, skin lesions	<1				
Togaviridae	+ssRNA	*Alphavirus*	CHIKV	Human, NHP, rodent, arthropod	Arthropod (mosquito)	febrile, polyarthralgia, multiorgan dysfunction	<1	X			(87-90)
			RRV	Marsupials, arthropod	Arthropod (mosquito)	febrile, polyarthralgia	<1				
			MAYV	NHP, arthropod	Arthropod (mosquito)	febrile, polyarthralgia	<1				
			ONNV	Human	Arthropod (mosquito)	febrile, polyarthralgia	<1				
			WEEV	Bird, arthropod	Arthropod (mosquito)	febrile, neurological (sequelae)	3 - 15				
			EEEV	Bird, rodent, arthropod	Arthropod (mosquito)	febrile, neurological (sequelae)	30 - 75				
			VEEV	Rodent, arthropod	Arthropod (mosquito)	febrile, neurological (sequelae)	<1				

Abbreviations: LCMV-lymphocytic choriomeningitis virus, LASV-Lassa virus, HCoV-human coronavirus, SARS-CoV-severe acute respiratory syndrome coronavirus, MERS-CoV-Middle East respiratory syndrome coronavirus, EBOV-Zaire ebolavirus, SUDV-Sudan ebolavirus, BDBV-Bundibugyo ebolavirus, MARV-Marburg virus, DENV-dengue virus, YFV-yellow fever virus, ZIKV-Zika virus, JEV-Japanese encephalitis virus, WNV-West Nile virus, TBEV-tick borne encephalitis virus, HCV-hepatitis C virus, HTNV-Hantaan virus, AMRV-Amur virus, DOBV-Dobrava Belgrade virus, CCHFV-Crimean Congo haemorrhagic fever virus, BJNV-Beiji nairovirus, IAV-influenza A virus, IBV-influenza B virus, NIV-Nipah virus, HEV-Hendra virus, MEV-measles virus, MUV-mumps virus, OROV-Oropouche virus, LACV-La Crosse virus, BUNV-Bunyamwera virus, RVFV-Rift Valley fever virus, TOSV-Toscana virus, HRTV-Heartland virus, SFTSV- severe fever with

thrombocytopenia syndrome virus, PV-poliovirus, RV-rhinovirus, EV-enterovirus, ECHOV-echovirus, CV-cardiovirus, SAFV-Saffold virus, VACV-vaccinia virus, MPX-Mpox virus, ORFV-ORF virus, YMTV-Yaba monkey tumor virus, TANV-Tanapox virus, CHIKV-chikungunya virus, RRV-Ross River virus, MAYV-Mayaro virus, ONNV-o'nyong nyong virus, WEEV-western equine encephalitis virus, EEEV-eastern equine encephalitis virus, VEEV-Venezuelan equine encephalitis virus, NHP-non-human primate, CFR-case-fatality rate

Supplementary Table S2. Datasets used for analysis

Family	Virus	Screen no.	Strain	Screen Type	Cell Line	Readout	Significance	Ranking	Ref
Arenaviridae	LCMV	1	Armstrong 53b	CRISPR	A549	Reporter virus	p ≤ 0.01	MAGeCK score	(91)
Coronaviridae	SARS-CoV-2	2	USA/WA-1	CRISPR	Huh7.5.1-ACE2/TMPRSS2	Cell survival	p ≤ 0.01	MAGeCK score	(92)
		3	France/IDF0372/2020	CRISPR	Vero	Cell survival	Z-score ≥ 2	Z-score	(93)
		4	France/IDF0372/2020	CRISPR	Calu3	Cell survival	Z-score ≥ 2	Z-score	(93)
		5	France/IDF0372/2020	CRISPR	Caco2-ACE2	Cell survival	Z-score ≥ 2	Z-score	(93)
		6	USA-WA1/2020	CRISPR	Huh7.5-ACE2/TMPRSS2	Cell survival	p ≤ 0.01	Z-score	(94)
		7	USA-WA1/2020	CRISPR	Huh7.5-ACE2/TMPRSS2	Cell survival	p ≤ 0.01	Z-score	(94)
		8	USA/WA-1	CRISPR	Calu3	Cell survival	FDR ≤ 0.05	MAGeCK score	(95)
		9	USA-WA1/2020	CRIPSR	hESCs	Cell survival	p ≤ 0.01	CRISPR score	(95)
		10	Singapore/2/2020	CRISPR	IGROV-1	Cell survival	p ≤ 0.01	MAGeCK score	(96)
		11	USA-WA1/2020	CRISPR	A549-ACE2	Cell survival	p ≤ 0.01	MAGeCK score	(97)
		12	HKU-001a	shRNA	HK-2	Cell survival	-	Fold change	(98)
		13	USA/WA-1	CRISPR	Calu3	Cell survival	FDR ≤ 0.1	MAGeCK score	(99)
		14	USA/WA-1	CRISPR	Vero	Cell survival	p ≤ 0.01	MAGeCK score	(100)
		15	USA/WA-1	CRISPR	Vero	Cell survival	Z-score ≥ 2	Z-score	(101)
	MERS	16	EMC	CRISPR	Huh7	Cell survival	p ≤ 0.01	MAGeCK score	(102)
		17	EMC/2012	CRISPR	Vero	Cell survival	Z-score ≥ 2	Z-score	(101)
	HCoV-229E	18	-	CRISPR	Huh7.5.1	Cell survival	p ≤ 0.01	MAGeCK score	(92)
		19	-	CRISPR	Huh7	Cell survival	p ≤ 0.01	MAGeCK score	(102)
		20	-	CRISPR	Huh7	Cell survival	p ≤ 0.01	MAGeCK score	(103)
		21	-	CRISPR	Huh7.5-ACE2-TMPRSS2	Cell survival	p ≤ 0.01	Z-score	(94)
	HCoV-OC43	22	-	CRISPR	Huh7.5.1	Cell survival	p ≤ 0.01	MAGeCK score	(92)
		23	-	CRISPR	Huh7.5-ACE2-TMPRSS2	Cell survival	p ≤ 0.01	Z-score	(94)
		24	-	CRISPR	IGROV-1	Cell survival	p ≤ 0.01	MAGeCK score	(96)
	HCoV-NL63	25	-	CRISPR	Huh7.5-ACE2-TMPRSS2	Cell survival	p ≤ 0.01	Z-score	(94)
Filoviridae	EBOV	26	Mayinga	CRISPR	Huh7.5.1	Cell survival	p ≤ 0.01	MAGeCK score	(104)
Flavivirus	HCV	27	JFH-1	siRNA	Huh7.5.1	Virus staining	-	%infected cells	(105)
		28	-	CRISPR	Huh7.5-NlrD	Reporter cell line	p ≤ 0.01	RIGER score	(106)
	ZIKV	29	MR766	CRISPR	H1-HeLa	Cell survival	p ≤ 0.01	#sgRNA and #reads	(107)
		30	BeH819015	CRISPR	human glioblastoma stem cells	Reporter virus	≥ 5 million reads	STARS score	(108)
		31	BeH819015	CRISPR	HEK293T	Reporter virus	≥ 5 million reads + ≥ 2 sgRNAs	p-value	(108)
	DENV	32	JAM	CRISPR	HAP1	Cell survival	p ≤ 0.01	MAGeCK score	(109)
		33	NGC	siRNA	HeLa-MAGI	Virus staining	p ≤ 0.01	p-value	(107)
	WNV	34	2741	siRNA	HeLa	Virus staining	p ≤ 0.01	Fold change	(110)
		35	B956	CRISPR	293FT	Cell survival	-	#sgRNA and #reads	(111)
		36	New York 2000	CRISPR	293T	Cell survival	p ≤ 0.01	MAGeCK score	(112)
	YFV	37	17D	siRNA	Huh7	Virus staining	p ≤ 0.01	Sum rank	(113)
Orthomyxoviridae	IAV	38	Puerto Rico/8/1934 H1N1	CRISPR	A549	Virus staining	p ≤ 0.01	p-value	(114)
		39	Vietnam/ 1203/04 H5N1	CRISPR	A549	Cell survival	p ≤ 0.01	MAGeCK score	(115)
		40	chicken/Guangxi/97/2017 H7N9	CRISPR	A549	Cell survival	p ≤ 0.01	MAGeCK score	(116)
		41	Anhui/1/2005 H5N1	CRISPR	A549	Cell survival	-	#reads	(117)
		42	WSN/33 H1N1	siRNA	A549	Reporter cell line	Z-score ≤ -2	Z-score	(118)
		43	PuertoRico/8/1934 H1N1	CRISPR	HEK293SF	Reporter virus	p ≤ 0.01	MAGeCK score	(119)
Paramyxoviridae	HEV	44	horse/1994	siRNA	HeLa	Reporter virus	Z-score ≤ -2	Z-score	(120)
	MEV	45	Khartoum-Sudan	siRNA	A549-hSLAM	Reporter virus	Z-score ≤ 2	Z-score	(121)
	MUV	46	G09 (New York)	siRNA	A549-hSLAM	Reporter virus	Z-score ≤ -2	Z-score	(121)
Phenuiviridae	HRTV	47	MO4	CRISPR	293T	Cell survival	p ≤ 0.01	MAGeCK score	(122)
	RVFV	48	MP12	siRNA	HeLa	Reporter virus	SSMD ≤-1.3	SSMD	(123)

Picornaviridae	EV71	49	5865/SIN/000009	siRNA	RD	Virus staining	Z-score ≤ -2	Z-score	(124)
		50	-	CRISPR	HeLa	Cell survival	p ≤ 0.01	MAGeCK score	(125)
	EVD68	51	Missouri	CRISPR	H1-Hela-CHDR3	Cell survival	p ≤ 0.01	MAGeCK score	(126)
	HAV	52	HM175/18f	CRISPR	Huh7.5.1	Cell survival	p ≤ 0.01	MAGeCK score	(127)
	PV	53	Sabin-2	siRNA	HepG2	ELISA	Z-score ≤ -2	Z-score	(128)
	RVB	54	B14	CRISPR	H1-HeLa	Cell survival	p ≤ 0.01	MAGeCK score	(129)
	RVC	55	C15	CRISPR	H1-Hela-CHDR3	Cell survival	p ≤ 0.01	MAGeCK score	(126)
Poxviridae	VACV	56	MVA	Gene trap	HAP1	Reporter virus	p ≤ 0.01	p-value	(130)
	MPXV	57	WA	Gene trap	HAP1	Cell survival	p ≤ 0.01	p-value	(131)
		58	ROC	Gene trap	HAP1	Cell survival	p ≤ 0.01	p-value	(131)
Togaviridae	CHIKV	59	181/25	CRISPR	3T3	Cell survival	p ≤ 0.01	MAGeCK score	(132)
		60	-	siRNA	HEK293	Reporter virus	cSSMD	SSMD	(133)
	SINV	61	-	siRNA	U2OS	Reporter virus	-	Z-score	(134)
		62	-	CRISPR	HCT116	Reporter virus	p ≤ 0.01	MAGeCK score	(135)

References

1. Radoshitzky SR, Buchmeier MJ, Charrel RN, Gonzalez JJ, Gunther S, Hepojoki J, et al. ICTV Virus Taxonomy Profile: Arenaviridae 2023. J Gen Virol. 2023;104(9).
2. Charrel RN, de Lamballerie X. Zoonotic aspects of arenavirus infections. Vet Microbiol. 2010;140(3-4):213–20.
3. Ajayi NA, Nwigwe CG, Azuogu BN, Onyire BN, Nwonwu EU, Ogbonnaya LU, et al. Containing a Lassa fever epidemic in a resource-limited setting: outbreak description and lessons learned from Abakaliki, Nigeria (January-March 2012). Int J Infect Dis. 2013;17(11):e1011–6.
4. Saleh M, Dan-Nwafor C, Ipadeola O, Ukponu W, Mba S, Abejegah C, et al. Exposure Incidents and Outcome of Lassa Fever Virus (LASV) Infection among Healthcare Workers in Nigeria, 2019 Journal of Infectious Diseases and Epidemiology. 2020;6(168).
5. Zapata JC, Pauza CD, Djavani MM, Rodas JD, Moshkoff D, Bryant J, et al. Lymphocytic choriomeningitis virus (LCMV) infection of macaques: a model for Lassa fever. Antiviral Res. 2011;92(2):125–38.
6. Vilibic-Cavlek T, Savic V, Ferenc T, Mrzljak A, Barbic L, Bogdanic M, et al. Lymphocytic Choriomeningitis-Emerging Trends of a Neglected Virus: A Narrative Review. Trop Med Infect Dis. 2021;6(2).
7. Garry RF. Lassa fever - the road ahead. Nat Rev Microbiol. 2023;21(2):87–96.
8. Woo PCY, de Groot RJ, Haagmans B, Lau SKP, Neuman BW, Perlman S, et al. ICTV Virus Taxonomy Profile: Coronaviridae 2023. J Gen Virol. 2023;104(4).
9. Gaunt ER, Hardie A, Claas EC, Simmonds P, Templeton KE. Epidemiology and clinical presentations of the four human coronaviruses 229E, HKU1, NL63, and OC43 detected over 3 years using a novel multiplex real-time PCR method. J Clin Microbiol. 2010;48(8):2940–7.
10. de Wit E, van Doremalen N, Falzarano D, Munster VJ. SARS and MERS: recent insights into emerging coronaviruses. Nat Rev Microbiol. 2016;14(8):523–34.
11. World Health Organization. WHO COVID-19 Dashboard - Cases [Available from: data.who.int/dashboards/covid19/cases.
12. World Health Organization. WHO COVID-19 Dashboard - Deaths [Available from: https://data.who.int/dashboards/covid19/deaths.
13. Alimohamadi Y, Tola HH, Abbasi-Ghahramanloo A, Janani M, Sepandi M. Case fatality rate of COVID-19: a systematic review and meta-analysis. J Prev Med Hyg. 2021;62(2):E311–E20.
14. Liu J, Wei H, He D. Differences in case-fatality-rate of emerging SARS-CoV-2 variants. Public Health Pract (Oxf). 2023;5:100350.
15. Riedmann U, Chalupka A, Richter L, Sprenger M, Rauch W, Krause R, et al. COVID-19 case fatality rate and infection fatality rate from 2020 to 2023: Nationwide analysis in Austria. J Infect Public Health. 2025;18(4):102698.
16. Xia Q, Yang Y, Wang F, Huang Z, Qiu W, Mao A. Case fatality rates of COVID-19 during epidemic periods of variants of concern: A meta-analysis by continents. Int J Infect Dis. 2024;141:106950.
17. Mohamed K, Rzymski P, Islam MS, Makuku R, Mushtaq A, Khan A, et al. COVID-19 vaccinations: The unknowns, challenges, and hopes. J Med Virol. 2022;94(4):1336–49.
18. Singh M, de Wit E. Antiviral agents for the treatment of COVID-19: Progress and challenges. Cell Rep Med. 2022;3(3):100549.
19. Kortepeter MG, Bausch DG, Bray M. Basic clinical and laboratory features of filoviral hemorrhagic fever. J Infect Dis. 2011;204 Suppl 3:S810–6.
20. Yamaoka S, Ebihara H. Pathogenicity and Virulence of Ebolaviruses with Species- and Variant-specificity. Virulence. 2021;12(1):885–901.
21. Cuomo-Dannenburg G, McCain K, McCabe R, Unwin HJT, Doohan P, Nash RK, et al. Marburg virus disease outbreaks, mathematical models, and disease parameters: a systematic review. Lancet Infect Dis. 2024;24(5):e307–e17.

22. Jacob ST, Crozier I, Fischer WA, 2nd, Hewlett A, Kraft CS, Vega MA, et al. Ebola virus disease. Nat Rev Dis Primers. 2020;6(1):13.
23. Miraglia CM. Marburgviruses: An Update. Lab Med. 2019;50(1):16–28.
24. Schuh AJ, Amman BR, Towner JS. Filoviruses and bats. Microbiol Aust. 2017;38(1):12–6.
25. Marzi A, Feldmann H. Filovirus vaccines as a response paradigm for emerging infectious diseases. NPJ Vaccines. 2024;9(1):186.
26. Taki E, Ghanavati R, Navidifar T, Dashtbin S, Heidary M, Moghadamnia M. Ebanga: The most recent FDA-approved drug for treating Ebola. Front Pharmacol. 2023;14:1083429.
27. Hartlage AS, Cullen JM, Kapoor A. The Strange, Expanding World of Animal Hepaciviruses. Annu Rev Virol. 2016;3(1):53–75.
28. Simmonds P, Becher P, Bukh J, Gould EA, Meyers G, Monath T, et al. ICTV Virus Taxonomy Profile: Flaviviridae. J Gen Virol. 2017;98(1):2–3.
29. Pierson TC, Diamond MS. The continued threat of emerging flaviviruses. Nat Microbiol. 2020;5(6):796–812.
30. Cheng Y, Tran Minh N, Tran Minh Q, Khandelwal S, Clapham HE. Estimates of Japanese Encephalitis mortality and morbidity: A systematic review and modeling analysis. PLoS Negl Trop Dis. 2022;16(5):e0010361.
31. Guo C, Zhou Z, Wen Z, Liu Y, Zeng C, Xiao D, et al. Global Epidemiology of Dengue Outbreaks in 1990-2015: A Systematic Review and Meta-Analysis. Front Cell Infect Microbiol. 2017;7:317.
32. Halani S, Tombindo PE, O'Reilly R, Miranda RN, Erdman LK, Whitehead C, et al. Clinical manifestations and health outcomes associated with Zika virus infections in adults: A systematic review. PLoS Negl Trop Dis. 2021;15(7):e0009516.
33. MacDonald RD, Krym VF. West Nile virus. Primer for family physicians. Can Fam Physician. 2005;51(6):833–7.
34. Tomori O. Yellow fever: the recurring plague. Crit Rev Clin Lab Sci. 2004;41(4):391–427.
35. Chiffi G, Grandgirard D, Leib SL, Chrdle A, Ruzek D. Tick-borne encephalitis: A comprehensive review of the epidemiology, virology, and clinical picture. Rev Med Virol. 2023;33(5):e2470.
36. Dutta SK, Langenburg T. A Perspective on Current Flavivirus Vaccine Development: A Brief Review. Viruses. 2023;15(4).
37. Geddawy A, Ibrahim YF, Elbahie NM, Ibrahim MA. Direct Acting Anti-hepatitis C Virus Drugs: Clinical Pharmacology and Future Direction. J Transl Int Med. 2017;5(1):8–17.
38. Avsic-Zupanc T, Saksida A, Korva M. Hantavirus infections. Clin Microbiol Infect. 2019;21S:e6–e16.
39. Afzal S, Ali L, Batool A, Afzal M, Kanwal N, Hassan M, et al. Hantavirus: an overview and advancements in therapeutic approaches for infection. Front Microbiol. 2023;14:1233433.
40. Kuhn JH, Alkhovsky SV, Avsic-Zupanc T, Bergeron E, Burt F, Ergunay K, et al. ICTV Virus Taxonomy Profile: Nairoviridae 2024. J Gen Virol. 2024;105(4).
41. Belobo JTE, Kenmoe S, Kengne-Nde C, Emoh CPD, Bowo-Ngandji A, Tchatchouang S, et al. Worldwide epidemiology of Crimean-Congo Hemorrhagic Fever Virus in humans, ticks and other animal species, a systematic review and meta-analysis. PLoS Negl Trop Dis. 2021;15(4):e0009299.
42. Hawman DW, Feldmann H. Crimean-Congo haemorrhagic fever virus. Nat Rev Microbiol. 2023;21(7):463–77.
43. Gargili A, Estrada-Pena A, Spengler JR, Lukashev A, Nuttall PA, Bente DA. The role of ticks in the maintenance and transmission of Crimean-Congo hemorrhagic fever virus: A review of published field and laboratory studies. Antiviral Res. 2017;144:93–119.
44. Spengler JR, Bergeron E, Rollin PE. Seroepidemiological Studies of Crimean-Congo Hemorrhagic Fever Virus in Domestic and Wild Animals. PLoS Negl Trop Dis. 2016;10(1):e0004210.

45. Wang YC, Wei Z, Lv X, Han S, Wang Z, Fan C, et al. A new nairo-like virus associated with human febrile illness in China. Emerg Microbes Infect. 2021;10(1):1200–8.
46. Bi Y, Yang J, Wang L, Ran L, Gao GF. Ecology and evolution of avian influenza viruses. Curr Biol. 2024;34(15):R716–R21.
47. Javanian M, Barary M, Ghebrehewet S, Koppolu V, Vasigala V, Ebrahimpour S. A brief review of influenza virus infection. J Med Virol. 2021;93(8):4638–46.
48. Iuliano AD, Roguski KM, Chang HH, Muscatello DJ, Palekar R, Tempia S, et al. Estimates of global seasonal influenza-associated respiratory mortality: a modelling study. Lancet. 2018;391(10127):1285–300.
49. Neumann G. H5N1 influenza virulence, pathogenicity and transmissibility: what do we know? Future Virol. 2015;10(8):971–80.
50. Sivanandy P, Zi Xien F, Woon Kit L, Tze Wei Y, Hui En K, Chia Lynn L. A review on current trends in the treatment of human infection with H7N9-avian influenza A. J Infect Public Health. 2019;12(2):153–8.
51. Taaffe J, Zhong S, Goldin S, Rawlings KS, Cowling BJ, Zhang W. An overview of influenza H5 vaccines. Lancet Respir Med. 2025;13(4):e20–e1.
52. Taaffe J, Ostrowsky JT, Mott J, Goldin S, Friede M, Gsell P, et al. Advancing influenza vaccines: A review of next-generation candidates and their potential for global health impact. Vaccine. 2024;42(26):126408.
53. Jones JC, Yen HL, Adams P, Armstrong K, Govorkova EA. Influenza antivirals and their role in pandemic preparedness. Antiviral Res. 2023;210:105499.
54. Kaza B, Aguilar HC. Pathogenicity and virulence of henipaviruses. Virulence. 2023;14(1):2273684.
55. Chua KB. Nipah virus outbreak in Malaysia. J Clin Virol. 2003;26(3):265–75.
56. Hossain MJ, Gurley ES, Montgomery JM, Bell M, Carroll DS, Hsu VP, et al. Clinical presentation of nipah virus infection in Bangladesh. Clin Infect Dis. 2008;46(7):977–84.
57. Hegde ST, Sazzad HM, Hossain MJ, Alam MU, Kenah E, Daszak P, et al. Investigating Rare Risk Factors for Nipah Virus in Bangladesh: 2001-2012. Ecohealth. 2016;13(4):720–8.
58. NSW Health. Summary of human cases of Hendra virus infection [updated 28 March 2022. Available from: https://www.health.nsw.gov.au/Infectious/controlguideline/Pages/hendra-case-summary.aspx.
59. Rima B, Balkema-Buschmann A, Dundon WG, Duprex P, Easton A, Fouchier R, et al. ICTV Virus Taxonomy Profile: Paramyxoviridae. J Gen Virol. 2019;100(12):1593–4.
60. Russell CJ, Simoes EAF, Hurwitz JL. Vaccines for the Paramyxoviruses and Pneumoviruses: Successes, Candidates, and Hurdles. Viral Immunol. 2018;31(2):133–41.
61. Hughes HR, Adkins S, Alkhovskiy S, Beer M, Blair C, Calisher CH, et al. ICTV Virus Taxonomy Profile: Peribunyaviridae. J Gen Virol. 2020;101(1):1–2.
62. Salvato RS. Re-emergence of Oropouche virus as a novel global threat. Curr Res Microb Sci. 2025;8:100406.
63. Vahey GM, Lindsey NP, Staples JE, Hills SL. La Crosse Virus Disease in the United States, 2003-2019. Am J Trop Med Hyg. 2021;105(3):807–12.
64. Dutuze MF, Nzayirambaho M, Mores CN, Christofferson RC. A Review of Bunyamwera, Batai, and Ngari Viruses: Understudied Orthobunyaviruses With Potential One Health Implications. Front Vet Sci. 2018;5:69.
65. Briese T, Bird B, Kapoor V, Nichol ST, Lipkin WI. Batai and Ngari viruses: M segment reassortment and association with severe febrile disease outbreaks in East Africa. J Virol. 2006;80(11):5627–30.
66. Sasaya T, Palacios G, Briese T, Di Serio F, Groschup MH, Neriya Y, et al. ICTV Virus Taxonomy Profile: Phenuiviridae 2023. J Gen Virol. 2023;104(9).
67. Ebogo-Belobo JT, Kenmoe S, Abanda NN, Bowo-Ngandji A, Mbaga DS, Magoudjou-Pekam JN, et al. Contemporary epidemiological data of Rift Valley fever virus in humans,

mosquitoes and other animal species in Africa: A systematic review and meta-analysis. Vet Med Sci. 2023;9(5):2309–28.
68. Nair N, Osterhaus A, Rimmelzwaan GF, Prajeeth CK. Rift Valley Fever Virus-Infection, Pathogenesis and Host Immune Responses. Pathogens. 2023;12(9).
69. Sui L, Sun P, Liu N, Zhao Y, Zhao Y, Zheng J, et al. The public health threat of emerging phenuiviruses. One Health. 2025;20:101055.
70. Keskek Turk Y, Ergunay K, Kohl A, Hughes J, McKimmie CS. Toscana virus - an emerging Mediterranean arbovirus transmitted by sand flies. J Gen Virol. 2024;105(11).
71. Kobayashi Y, Kato H, Yamagishi T, Shimada T, Matsui T, Yoshikawa T, et al. Severe Fever with Thrombocytopenia Syndrome, Japan, 2013-2017. Emerg Infect Dis. 2020;26(4):692–9.
72. Lee M, Lee E, Kim SW, Kim YK, Bae IG, Kim J, et al. Severe Fever with Thrombocytopenia Syndrome in South Korea, 2016-2021: Clinical Features of Severe Progression and Complications. Am J Trop Med Hyg. 2024;111(3):661–70.
73. Zhang XA, Li H, Jiang FC, Zhu F, Zhang YF, Chen JJ, et al. A Zoonotic Henipavirus in Febrile Patients in China. N Engl J Med. 2022;387(5):470–2.
74. Feng K, Bendiwhobel Ushie B, Zhang H, Li S, Deng F, Wang H, et al. Pathogenesis and virulence of Heartland virus. Virulence. 2024;15(1):2348252.
75. Nathanson N, Kew OM. From emergence to eradication: the epidemiology of poliomyelitis deconstructed. Am J Epidemiol. 2010;172(11):1213–29.
76. Link-Gelles R, Lutterloh E, Schnabel Ruppert P, Backenson PB, St George K, Rosenberg ES, et al. Public Health Response to a Case of Paralytic Poliomyelitis in an Unvaccinated Person and Detection of Poliovirus in Wastewater - New York, June-August 2022. MMWR Morb Mortal Wkly Rep. 2022;71(33):1065–8.
77. Jartti M, Flodstrom-Tullberg M, Hankaniemi MM. Enteroviruses: epidemic potential, challenges and opportunities with vaccines. J Biomed Sci. 2024;31(1):73.
78. Rao CD. Enteroviruses in gastrointestinal diseases. Rev Med Virol. 2021;31(1):1–12.
79. Ma E, Chan KC, Cheng P, Wong C, Chuang SK. The enterovirus 71 epidemic in 2008--public health implications for Hong Kong. Int J Infect Dis. 2010;14(9):e775–80.
80. Tan SZ, Tan MZ, Prabakaran M. Saffold virus, an emerging human cardiovirus. Rev Med Virol. 2017;27(1).
81. Srinivasan Rajsri K, Rao M. Poxvirus-driven human diseases and emerging therapeutics. Ther Adv Infect Dis. 2022;9:20499361221136751.
82. Sklenovska N, Van Ranst M. Emergence of Monkeypox as the Most Important Orthopoxvirus Infection in Humans. Front Public Health. 2018;6:241.
83. Billioux BJ, Mbaya OT, Sejvar J, Nath A. Potential complications of monkeypox. Lancet Neurol. 2022;21(10):872.
84. Lu J, Xing H, Wang C, Tang M, Wu C, Ye F, et al. Mpox (formerly monkeypox): pathogenesis, prevention, and treatment. Signal Transduct Target Ther. 2023;8(1):458.
85. Petersen E, Kantele A, Koopmans M, Asogun D, Yinka-Ogunleye A, Ihekweazu C, et al. Human Monkeypox: Epidemiologic and Clinical Characteristics, Diagnosis, and Prevention. Infect Dis Clin North Am. 2019;33(4):1027–43.
86. Islam MR, Hossain MJ, Roy A, Hasan A, Rahman MA, Shahriar M, et al. Repositioning potentials of smallpox vaccines and antiviral agents in monkeypox outbreak: A rapid review on comparative benefits and risks. Health Sci Rep. 2022;5(5):e798.
87. Chen R, Mukhopadhyay S, Merits A, Bolling B, Nasar F, Coffey LL, et al. ICTV Virus Taxonomy Profile: Togaviridae. J Gen Virol. 2018;99(6):761–2.
88. de Souza WM, Lecuit M, Weaver SC. Chikungunya virus and other emerging arthritogenic alphaviruses. Nat Rev Microbiol. 2025.
89. Woodson CM, Carney SK, Kehn-Hall K. Neuropathogenesis of Encephalitic Alphaviruses in Non-Human Primate and Mouse Models of Infection. Pathogens. 2025;14(2).

90. Richardson JS, Anderson DM, Mendy J, Tindale LC, Muhammad S, Loreth T, et al. Chikungunya virus virus-like particle vaccine safety and immunogenicity in adolescents and adults in the USA: a phase 3, randomised, double-blind, placebo-controlled trial. Lancet. 2025;405(10487):1343–52.
91. Liu J, Knopp KA, Rackaityte E, Wang CY, Laurie MT, Sunshine S, et al. Genome-Wide Knockout Screen Identifies Human Sialomucin CD164 as an Essential Entry Factor for Lymphocytic Choriomeningitis Virus. mBio. 2022;13(3):e0020522.
92. Wang R, Simoneau CR, Kulsuptrakul J, Bouhaddou M, Travisano KA, Hayashi JM, et al. Genetic Screens Identify Host Factors for SARS-CoV-2 and Common Cold Coronaviruses. Cell. 2021;184(1):106–19 e14.
93. Rebendenne A, Roy P, Bonaventure B, Chaves Valadao AL, Desmarets L, Arnaud-Arnould M, et al. Bidirectional genome-wide CRISPR screens reveal host factors regulating SARS-CoV-2, MERS-CoV and seasonal HCoVs. Nat Genet. 2022;54(8):1090–102.
94. Schneider WM, Luna JM, Hoffmann HH, Sanchez-Rivera FJ, Leal AA, Ashbrook AW, et al. Genome-Scale Identification of SARS-CoV-2 and Pan-coronavirus Host Factor Networks. Cell. 2021;184(1):120–32 e14.
95. Zhu S, Liu Y, Zhou Z, Zhang Z, Xiao X, Liu Z, et al. Genome-wide CRISPR activation screen identifies candidate receptors for SARS-CoV-2 entry. Sci China Life Sci. 2022;65(4):701–17.
96. Yousefi M, Lee WS, Chan WOY, He W, Mah MG, Yong CL, et al. Betacoronaviruses SARS-CoV-2 and HCoV-OC43 infections in IGROV-1 cell line require aryl hydrocarbon receptor. Emerg Microbes Infect. 2023;12(2):2256416.
97. Hou J, Wei Y, Zou J, Jaffery R, Sun L, Liang S, et al. Integrated multi-omics analyses identify anti-viral host factors and pathways controlling SARS-CoV-2 infection. Nat Commun. 2024;15(1):109.
98. Yeung ML, Teng JLL, Jia L, Zhang C, Huang C, Cai JP, et al. Soluble ACE2-mediated cell entry of SARS-CoV-2 via interaction with proteins related to the renin-angiotensin system. Cell. 2021;184(8):2212–28 e12.
99. Biering SB, Sarnik SA, Wang E, Zengel JR, Leist SR, Schafer A, et al. Genome-wide bidirectional CRISPR screens identify mucins as host factors modulating SARS-CoV-2 infection. Nat Genet. 2022;54(8):1078–89.
100. Mao D, Liu S, Phan AT, Renner S, Sun Y, Wang TT, et al. The TRAF3-DYRK1A-RAD54L2 complex maintains ACE2 expression to promote SARS-CoV-2 infection. J Virol. 2024;98(5):e0034724.
101. Wei J, Alfajaro MM, DeWeirdt PC, Hanna RE, Lu-Culligan WJ, Cai WL, et al. Genome-wide CRISPR Screens Reveal Host Factors Critical for SARS-CoV-2 Infection. Cell. 2021;184(1):76–91 e13.
102. Kratzel A, Kelly JN, V'Kovski P, Portmann J, Bruggemann Y, Todt D, et al. A genome-wide CRISPR screen identifies interactors of the autophagy pathway as conserved coronavirus targets. PLoS Biol. 2021;19(12):e3001490.
103. Trimarco JD, Heaton BE, Chaparian RR, Burke KN, Binder RA, Gray GC, et al. TMEM41B is a host factor required for the replication of diverse coronaviruses including SARS-CoV-2. PLoS Pathog. 2021;17(5):e1009599.
104. Flint M, Chatterjee P, Lin DL, McMullan LK, Shrivastava-Ranjan P, Bergeron E, et al. A genome-wide CRISPR screen identifies N-acetylglucosamine-1-phosphate transferase as a potential antiviral target for Ebola virus. Nat Commun. 2019;10(1):285.
105. Li Q, Brass AL, Ng A, Hu Z, Xavier RJ, Liang TJ, et al. A genome-wide genetic screen for host factors required for hepatitis C virus propagation. Proc Natl Acad Sci U S A. 2009;106(38):16410–5.
106. Liang Y, Zhang G, Li Q, Han L, Hu X, Guo Y, et al. TRIM26 is a critical host factor for HCV replication and contributes to host tropism. Sci Adv. 2021;7(2).

107. Savidis G, McDougall WM, Meraner P, Perreira JM, Portmann JM, Trincucci G, et al. Identification of Zika Virus and Dengue Virus Dependency Factors using Functional Genomics. Cell Rep. 2016;16(1):232–46.
108. Wang S, Zhang Q, Tiwari SK, Lichinchi G, Yau EH, Hui H, et al. Integrin alphavbeta5 Internalizes Zika Virus during Neural Stem Cells Infection and Provides a Promising Target for Antiviral Therapy. Cell Rep. 2020;30(4):969–83 e4.
109. Labeau A, Simon-Loriere E, Hafirassou ML, Bonnet-Madin L, Tessier S, Zamborlini A, et al. A Genome-Wide CRISPR-Cas9 Screen Identifies the Dolichol-Phosphate Mannose Synthase Complex as a Host Dependency Factor for Dengue Virus Infection. J Virol. 2020;94(7).
110. Krishnan MN, Ng A, Sukumaran B, Gilfoy FD, Uchil PD, Sultana H, et al. RNA interference screen for human genes associated with West Nile virus infection. Nature. 2008;455(7210):242–5.
111. Ma H, Dang Y, Wu Y, Jia G, Anaya E, Zhang J, et al. A CRISPR-Based Screen Identifies Genes Essential for West-Nile-Virus-Induced Cell Death. Cell Rep. 2015;12(4):673–83.
112. Zhang R, Miner JJ, Gorman MJ, Rausch K, Ramage H, White JP, et al. A CRISPR screen defines a signal peptide processing pathway required by flaviviruses. Nature. 2016;535(7610):164–8.
113. Le Sommer C, Barrows NJ, Bradrick SS, Pearson JL, Garcia-Blanco MA. G protein-coupled receptor kinase 2 promotes flaviviridae entry and replication. PLoS Negl Trop Dis. 2012;6(9):e1820.
114. Li B, Clohisey SM, Chia BS, Wang B, Cui A, Eisenhaure T, et al. Genome-wide CRISPR screen identifies host dependency factors for influenza A virus infection. Nat Commun. 2020;11(1):164.
115. Han J, Perez JT, Chen C, Li Y, Benitez A, Kandasamy M, et al. Genome-wide CRISPR/Cas9 Screen Identifies Host Factors Essential for Influenza Virus Replication. Cell Rep. 2018;23(2):596–607.
116. Yi C, Cai C, Cheng Z, Zhao Y, Yang X, Wu Y, et al. Genome-wide CRISPR-Cas9 screening identifies the CYTH2 host gene as a potential therapeutic target of influenza viral infection. Cell Rep. 2022;38(13):110559.
117. Song Y, Huang H, Hu Y, Zhang J, Li F, Yin X, et al. A genome-wide CRISPR/Cas9 gene knockout screen identifies immunoglobulin superfamily DCC subclass member 4 as a key host factor that promotes influenza virus endocytosis. PLoS Pathog. 2021;17(12):e1010141.
118. Karlas A, Machuy N, Shin Y, Pleissner KP, Artarini A, Heuer D, et al. Genome-wide RNAi screen identifies human host factors crucial for influenza virus replication. Nature. 2010;463(7282):818–22.
119. Sharon DM, Nesdoly S, Yang HJ, Gelinas JF, Xia Y, Ansorge S, et al. A pooled genome-wide screening strategy to identify and rank influenza host restriction factors in cell-based vaccine production platforms. Sci Rep. 2020;10(1):12166.
120. Deffrasnes C, Marsh GA, Foo CH, Rootes CL, Gould CM, Grusovin J, et al. Genome-wide siRNA Screening at Biosafety Level 4 Reveals a Crucial Role for Fibrillarin in Henipavirus Infection. PLoS Pathog. 2016;12(3):e1005478.
121. Anderson DE, Pfeffermann K, Kim SY, Sawatsky B, Pearson J, Kovtun M, et al. Comparative Loss-of-Function Screens Reveal ABCE1 as an Essential Cellular Host Factor for Efficient Translation of Paramyxoviridae and Pneumoviridae. mBio. 2019;10(3).
122. Xia T, Wu X, Hong E, Jung K, Lai CJ, Kwak MJ, et al. Glucosylceramide is essential for Heartland and Dabie bandavirus glycoprotein-induced membrane fusion. PLoS Pathog. 2023;19(3):e1011232.
123. Harmon B, Bird SW, Schudel BR, Hatch AV, Rasley A, Negrete OA. A Genome-Wide RNA Interference Screen Identifies a Role for Wnt/beta-Catenin Signaling during Rift Valley Fever Virus Infection. J Virol. 2016;90(16):7084–97.

124. Wu KX, Phuektes P, Kumar P, Goh GY, Moreau D, Chow VT, et al. Human genome-wide RNAi screen reveals host factors required for enterovirus 71 replication. Nat Commun. 2016;7:13150.
125. Guo D, Yu X, Wang D, Li Z, Zhou Y, Xu G, et al. SLC35B2 Acts in a Dual Role in the Host Sulfation Required for EV71 Infection. J Virol. 2022;96(9):e0204221.
126. Diep J, Ooi YS, Wilkinson AW, Peters CE, Foy E, Johnson JR, et al. Enterovirus pathogenesis requires the host methyltransferase SETD3. Nat Microbiol. 2019;4(12):2523–37.
127. Kulsuptrakul J, Wang R, Meyers NL, Ott M, Puschnik AS. A genome-wide CRISPR screen identifies UFMylation and TRAMP-like complexes as host factors required for hepatitis A virus infection. Cell Rep. 2021;34(11):108859.
128. van der Sanden SM, Wu W, Dybdahl-Sissoko N, Weldon WC, Brooks P, O'Donnell J, et al. Engineering Enhanced Vaccine Cell Lines To Eradicate Vaccine-Preventable Diseases: the Polio End Game. J Virol. 2016;90(4):1694–704.
129. Mei H, Zha Z, Wang W, Xie Y, Huang Y, Li W, et al. Surfaceome CRISPR screen identifies OLFML3 as a rhinovirus-inducible IFN antagonist. Genome Biol. 2021;22(1):297.
130. Luteijn RD, van Diemen F, Blomen VA, Boer IGJ, Manikam Sadasivam S, van Kuppevelt TH, et al. A Genome-Wide Haploid Genetic Screen Identifies Heparan Sulfate-Associated Genes and the Macropinocytosis Modulator TMED10 as Factors Supporting Vaccinia Virus Infection. J Virol. 2019;93(13).
131. Realegeno S, Puschnik AS, Kumar A, Goldsmith C, Burgado J, Sambhara S, et al. Monkeypox Virus Host Factor Screen Using Haploid Cells Identifies Essential Role of GARP Complex in Extracellular Virus Formation. J Virol. 2017;91(11).
132. Zhang R, Kim AS, Fox JM, Nair S, Basore K, Klimstra WB, et al. Mxra8 is a receptor for multiple arthritogenic alphaviruses. Nature. 2018;557(7706):570–4.
133. Karlas A, Berre S, Couderc T, Varjak M, Braun P, Meyer M, et al. A human genome-wide loss-of-function screen identifies effective chikungunya antiviral drugs. Nat Commun. 2016;7:11320.
134. Ooi YS, Stiles KM, Liu CY, Taylor GM, Kielian M. Genome-wide RNAi screen identifies novel host proteins required for alphavirus entry. PLoS Pathog. 2013;9(12):e1003835.
135. Petitjean O, Girardi E, Ngondo RP, Lupashin V, Pfeffer S. Genome-Wide CRISPR-Cas9 Screen Reveals the Importance of the Heparan Sulfate Pathway and the Conserved Oligomeric Golgi Complex for Synthetic Double-Stranded RNA Uptake and Sindbis Virus Infection. mSphere. 2020;5(6).
